# Mesenchymal WNT signaling coordinates epithelial and mesenchymal differentiation in the developing murine ureter

**DOI:** 10.1186/s12964-026-03094-6

**Published:** 2026-07-21

**Authors:** Mark-Oliver Trowe, Philipp Straube, Florian Bergmann, Nurullah Aydogdu, Hauke Thiesler, Herbert Hildebrandt, Tobias Bohnenpoll, Andreas Kispert

**Affiliations:** 1 Hannover Medical School, Institute of Molecular Biology, Hannover, 30625 Germany; 2 Hannover Medical School, Institute of Clinical Biochemistry, Hannover, 30625 Germany; 3 Institut für Molekularbiologie, OE5250, Medizinische Hochschule Hannover, Carl-Neuberg-Str. 1, Hannover, D-30625 Germany

**Keywords:** Mouse embryo, Ureter, Smooth muscle cells, Urothelium, Cell differentiation, WNT/β-Catenin-signaling, Conditional mouse mutants

## Abstract

**Background:**

The coordinated development of the mesenchymal and epithelial primordia of the murine ureter requires intense tissue communication. Previous studies have revealed the crucial role of the FGFR2-SHH-FOXF1-BMP4 signaling axis in the proliferation and differentiation programs of both the epithelium and the mesenchyme. Canonical (β-Catenin/CTNNB1-dependent) WNT signaling in the mesenchymal primordium has been reported to influence mesenchymal development by enhancing proliferation and favoring the smooth muscle cell fate over adventitial fibrocytes.

**Methods:**

To investigate the role of mesenchymal WNT signaling in early ureter development further, we analyzed the ureters of *Ctnnb1*-deficient mouse mutants for cellular and molecular changes during embryogenesis. Using pharmacological inhibition and activation approaches in explant cultures of embryonic ureters, we assessed the contribution of altered signaling activities to differentiation changes in *Ctnnb1*-deficient ureters. Transcriptional profiling of embryonic ureters with short-term WNT signaling inhibition allowed us to identify the primary targets of this signaling pathway in the ureteric mesenchyme.

**Results:**

We demonstrate that both mesenchymal and epithelial differentiation are impaired in ureters lacking mesenchymal *Ctnnb1*. This defect is linked to an inability to activate pro-differentiation transcription factors in either tissue primordia. Epithelial changes can be attributed, at least in part, to the loss of BMP4 signaling and the gain of canonical WNT signaling in the epithelial primordium. Mesenchymal WNT signaling directly activates transcription of multiple components of the SHH-FOXF1-BMP4 signaling axis, thereby enhancing this module in the inner region of the ureteric mesenchyme.

**Conclusions:**

Our work improves the understanding of the signaling network that coordinates cytodifferentiation in the early ureter. Mesenchymal WNT signaling plays a central role in mesenchymal fate decisions but also in promoting epithelial differentiation through enhancement of the SHH-FOXF1-BMP4 axis and inhibition of epithelial WNT-signaling.

**Supplementary Information:**

The online version contains supplementary material available at 10.1186/s12964-026-03094-6.

## Introduction

The mammalian ureters are tubular organs for the efficient long-distance transport of urine from the kidneys to the bladder. They have an inner epithelial compartment called the urothelium, which consists of layers of basal (B), intermediate (I) and superficial (S) cells. S cells have tight junctions and express uroplakins (UPK) to establish a permeability barrier to the luminal content. The outer mesenchymal wall contains a thick layer of contractile smooth muscle cells (SMCs) called the tunica muscularis. This, along with the fibro-elastic material of the inner lamina propria and outer tunica adventitia, accounts for the tube’s peristaltic activity and flexible rigidity (Fig. S1A) [1, 2].

Defects in the structural integrity and/or functional activity of the SMC layer and the urothelium of the ureter prevent the efficient drainage of the urine from the renal pelvis to the bladder. Depending on the level and intensity of the structural or functional obstruction, urine may enter the interstitial space (efflux) and/or accumulate in and dilate the ureter (reflux). Hydroureter, as the latter condition is called, will lead to the dilation of the pelvis and collecting duct system of the kidney (hydronephrosis) and may progress to pressure-mediated destruction of the renal parenchyma [3, 4]. Congenital forms of these anomalies represent a prominent sub-group of congenital anomalies of the kidney and the urinary tract (CAKUT) that belong to the most frequent human birth defects [5–7]. They arise, at least in part, from genetic perturbation of the cellular processes that guide the development of the ureteric tissues. Since the characterization of the developmental etiology and molecular cause of hydroureter-nephrosis and other ureteral anomalies is difficult in humans, the experimental efforts to unravel the cellular processes and the molecular circuits that regulate normal and pathological growth and differentiation of the ureter have focused on the mouse [8].

Cell lineage and marker analyses in this model system have shown that the different epithelial and mesenchymal cell types of the ureter arise in a highly coordinated manner from embryonic day (E) 11.5 onwards from two distinct precursor tissues in the early metanephric field, namely the distal part of the ureteric bud (UB) - an epithelial outgrowth of the nephric duct (ND) - and the surrounding mesenchyme [9, 10]. From E11.5 to E14.5, the epithelial and mesenchymal progenitors strongly proliferate to elongate the primitive ureter tube. While the epithelial progenitors remain organized in a monolayer during that time, the surrounding mesenchymal cells become histologically separated into an inner layer of rhomboid-shaped cells and an outer layer which maintains the fibrocytic appearance. From E14.5 until birth, proliferation continues at a lower level to support further tubular elongation but also tissue expansion; differentiated cell types are gradually established. The ureteric epithelium (UE) stratifies into three cell layers, accompanied by the differentiation of I cells at E14.5, S cells at E16.5, and B cells at E18.5. The cells of the inner layer of the ureteric mesenchyme (UM) differentiate into SMCs from E15.5 onwards, preceded by the expression of the SMC regulatory factor Myocardin (MYOCD) at E14.5. After E16.5, some mesenchymal cells adjacent to the urothelium switch off the myogenic program and regain a fibrocytic phenotype. (For a scheme of layer formation and cell differentiation in embryonic and fetal ureter development see Fig. S1B). Through proliferation and the secretion of extracellular matrix (ECM), these cells will form an expanded lamina propria in the first postnatal week of life [9–11].

Genetic analysis has shown that the coordinated proliferation and entry of the ureteral tissue primordia into the cytodifferentiation programs depend on an FGFR2-SHH-FOXF1-BMP4 signaling module [8]. FGFR2 signaling enhances expression of *Shh* in the epithelial primordium [12]. Upon secretion, SHH activates the expression of the transcription factor (TF) gene *Foxf1* in the UM. FOXF1 then enhances expression of the signaling protein BMP4 [13]. BMP4 supports proliferation in both tissue primordia. Moreover, BMP4 acts *in trans* to activate the expression of TFs crucial for epithelial stratification and differentiation (such as *Trp63*) [14, 15], and *in cis* to activate expression of the TF gene *Gata6*, whose gene product cooperates with FOXF1 in activating *Myocd* expression and the SMC differentiation program [13, 15–17]. BMP4 signaling also suppresses the expression of *Aldh1a2*, a gene encoding a biosynthetic enzyme for retinoic acid (RA) in the UM, thereby reducing RA signaling and the anti-differentiation activity of this pathway [18, 19].

We have previously shown that canonical WNT signaling occurs in the inner layer of the UM from E11.5 to E14.5. Conditional (*Tbx18*^*cre*^-mediated) mesenchymal deletion of *Ctnnb1* (the gene encoding β-Catenin) - the key factor in the canonical sub-branch of WNT signaling – caused reduced proliferation of the inner region of the UM, abolished *Myocd* activation at E14.5 as well as subsequent SMC differentiation. This was accompanied by an expansion of the adventitial fate into the inner region of the UM around E14.5, increased collagen deposition in the mesenchymal wall and hydroureter formation at birth [20]. The closely related TFs TBX2 and TBX3 are expressed in the inner UM in a *Ctnnb1*-dependent manner and are responsible for restricting the adventitial fate to the outer UM [21].

How mesenchymal WNT signaling impinges on activation of *Myocd* expression and SMC differentiation, whether it interacts with one of the other signaling pathways regulating this process, and whether it has additional molecular and possibly cellular functions has remained enigmatic. Here, we used unbiased transcriptional profiling of ureters with genetic or pharmacological loss of canonical WNT signaling to further elucidate the molecular function of this pathway in early ureter development. Our results demonstrate that canonical WNT signaling in the UM plays a crucial role in the coordinated cytodifferentiation of the mesenchymal and epithelial tissue primordia of the ureter by suppressing epithelial WNT signaling and activating the SHH-FOXF1-BMP4 signaling axis in the inner layer of the UM.

## Results

### Complete absence of mesenchymal and epithelial cytodifferentiation in *Ctnnb1-cKO* ureters at prenatal stages

Our previous work has shown that in *Tbx18*^*cre/+*^;*Ctnnb1*^*fl/fl*^ (*Ctnnb1-cKO)* embryos, the development of the mesenchymal compartment of the ureter is severely disturbed: proliferation in the inner region of the UM is reduced at E12.5 and E14.5, *Myocd* is not activated at E14.5, and differentiated SMCs are completely absent at E18.5. We did not detect changes of proliferation and apoptosis in the epithelial primordium at E12.5 and E14.5 in that study, but found reduced expression of the S-cell specific gene *Upk3a* in the UE at E18.5 [20].

**Fig. 1:**
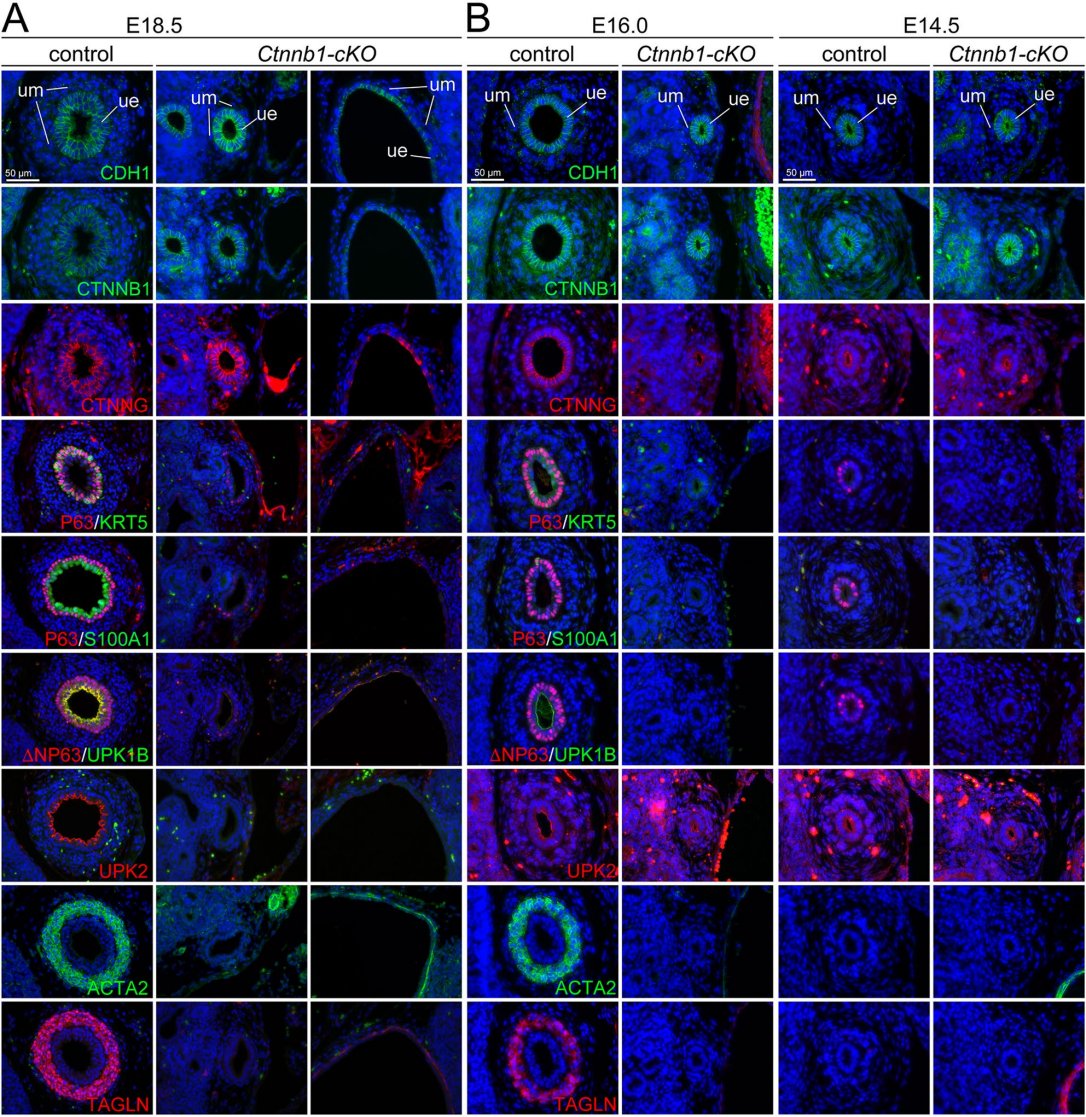
*Ctnnb1-cKO* ureters lack epithelial and mesenchymal cytodifferentiation. **A**,** B** Immunofluorescence analysis on proximal ureter sections from control and *Ctnnb1-KO* embryos at E18.5 (**A**), and E16.0 and E14.5 (**B**), for markers of epithelial stratification and adherence (CDH1, CTNNB1, CTNNG), differentiation of urothelial S cells (S100A1, UPK2), S and I cells (UPK1B), B and I cells (ΔNP63, P63) and B cells (KRT5), as well as smooth muscle cells (ACTA2, TAGLN). Please note that we used a rare undilated specimen in addition to one of the frequent dilated specimens for E18.5 *Ctnnb1-cKO* ureters. Please also note that the CTNNB1 signal is reduced in the UM but not in the UE of mutant embryos at E14.5 and E16.0. *n* = 4 per antibody and genotype. ue, ureteric epithelium; um, ureteric mesenchyme

To further explore E18.5 *Ctnnb1-cKO* ureters for defects in urothelial differentiation, we analyzed by immunofluorescence the expression of marker proteins indicative of epithelial integrity (adhesion and polarization), stratification and cytodifferentiation (Fig. 1A). CDH1 is a core component of adherens junctions in epithelial cells [22]. In the control ureter, CDH1 expression labelled the baso-lateral membrane of all cells in the inner (epithelial) compartment, which was organized into two to three layers. In the mutant ureter, the inner compartment was mono-layered but all cells invariably expressed CDH1 in the baso-lateral membrane, albeit at a possibly slightly increased level compared to the control - this is best seen in a rare specimen, which did not exhibit the massive dilatation typical for *Ctnnb1-cKO* ureters at this stage (Fig. 1A). We also analyzed expression of CTNNB1 and CTNNG (γ-Catenin, Plakoglobin), which anchor cadherins to the intracellular cytoskeleton [22]. Both proteins recapitulated the pattern of CDH1 expression in the cells of the mutant urothelium as in the control. Similar to CDH1, membraneous expression of CTNNG appeared increased in the mutant. Together this shows that the cells of the inner compartment of the *Ctnnb1-cKO* ureter are organized as an epithelial mono-layer, and that the structural integrity of adherens junctions may be partially affected (Fig. 1A).

Expression of KRT5 in the subcortical cytoplasm, ΔNP63 (an isoform of TRP63) in the nucleus, UPK1B/UPK2 in the cytoplasm and apical membrane, and S100A1 in the cytoplasm combinatorially marked B cells (KRT5^+^ΔNP63^+^UPK1B^−^UPK2^−^S100A1^−^), I cells (KRT5^−^ΔNP63^+^UPK1B^low^UPK2^low^S100A1^−^) and S cells (KRT5^−^ΔNP63^−^UPK1B^+^UPK2^+^S100A1^+^) in the control urothelium [9, 23]. In *Ctnnb1-cKO* ureters, the mono-layered epithelium lacked expression of KRT5, UPK1B, UPK2 and S100A1; the number of P63^+^ and ΔNP63^+^ cells was strongly reduced, indicating a rather complete lack of differentiated B, I and S cells (Fig. 1A). Markers for cell types of renal collecting ducts such as AQP2 (principal cells), and FOXI1 and ATP6V1B1/2 (intercalated cells) [24] were absent from the UE in *Ctnnb1-cKO* embryos (Fig. S2), ruling out a shift towards the epithelial differentiation program of the kidney. Consistent with our previous findings [20], we observed an absence of SMC markers (ACTA2 and TAGLN) in *Ctnnb1-cKO* ureters at E18.5 (Fig. 1A).

In normal ureters, expression of the epithelial and mesenchymal cytodifferentiation markers P63/ΔNP63, KRT5, UPK1B, UPK2 and S100A1 is gradually activated from E14.5 onwards. In *Ctnnb1-cKO* ureters, none of these markers was expressed at E14.5 and E16.0. CDH1 and CTNNB1 expression labelled the basolateral membranes of the epithelial cells as in the control, whereas expression of CTNNG appeared reduced in the mutant epithelium (Fig. 1B). We conclude that loss of *Ctnnb1*-dependent WNT signaling in the mesenchymal primordium of the ureter does not only affect SMC development as shown before [20], but also compromises the epithelial stratification and differentiation programs from E14.5 at least until birth. The establishment of adherence junctions may be delayed and/or partially affected.

### Mesenchymal WNT signaling is required for the expression of pro-differentiation TF genes in the epithelial and mesenchymal tissue primordium of the ureter

**Fig. 2:**
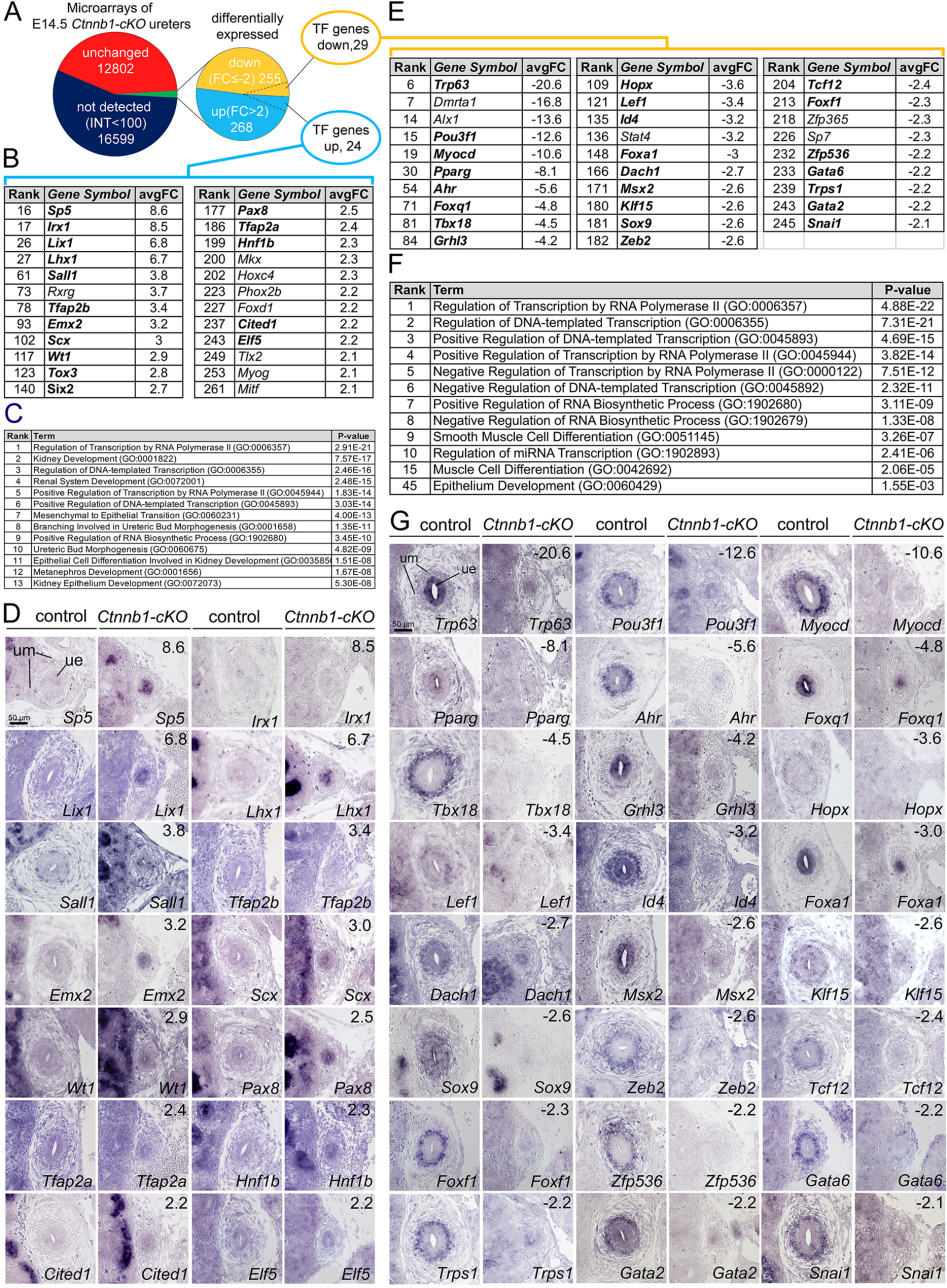
Mesenchymal WNT signaling regulates the expression of various TF genes in the E14.5 ureter. **A** Pie chart summarizing the results of the microarray analysis of two pools each of E14.5 control and *Ctnnb1-cKO* ureters. **B**,** E** List of 24 transcription factor (TF) genes with increased expression (**B**) and of 29 TF genes with reduced expression (**E**) in the E14.5 *Ctnnb1-cKO* ureter microarrays. Shown are the overall rank, the gene name and the average fold change (avgFC) of expression. **C**,** F** Gene ontology (GO) enrichment analysis for upregulated TF genes (**C**) and for downregulated TF genes (**F**). The top 10 scoring terms as well as selected others are listed with their *P*-value. **D**,** G** RNA in situ hybridization analysis on proximal ureter sections of E14.5 control and *Ctnnb1-cKO* embryos for expression of TF genes with increased expression in microarrays of E14.5 *Ctnnb1-cKO* ureters (marked in bold in **B**) and with reduced expression (marked in bold in **E**). Numbers in the upper right corner relate to the avgFC in the microarray. *n* = 3 per probe and genotype. ue, ureteric epithelium; um, ureteric mesenchyme

To unravel tissue-specific TFs, whose altered expression may underlie the cytodifferentiation defects in *Ctnnb1-cKO* ureters, we performed unbiased transcriptional profiling of control and mutant ureters at E14.5, a stage when the corresponding genes are normally activated [15, 18, 25]. Using cut-offs of intensities ≥ 100 and fold changes (FCs) ≥ 2 in each of the two microarrays to detect robust expression changes, we identified a total of 268 upregulated and 255 downregulated genes (Fig. 2A, Table S1 and S2).

Twenty-four of the 268 upregulated genes encoded tissue-specific TFs (Fig. 2B, Table S3). Annotation of biological processes using the subprogram “GO Biological Process 2025” within the web-based “Enrichr” gene list enrichment analysis tool (https://maayanlab.cloud/Enrichr/) [26–28] uncovered for this TF gene set numerous gene ontology (GO) terms related to “kidney development” (Fig. 2C, Table S4). In fact, many of these TFs have previously been implicated in ND, early UB and renal development [29, 30]. To determine the tissue-specificity and validate the upregulation of expression of those TF genes, for which we were able to generate suitable riboprobes, we performed RNA in situ hybridization analysis on proximal sections of E14.5 control and *Ctnnb1-cKO* ureters. Probably due to the low level of transcripts, we failed to detect reliable expression of some of the candidates (*Irx1*,* Tfap2b*, *Scx*,* Tfap2a*, *Wt1*,* Cited1* and *Elf5)* both in the control and mutant ureter. S*all1* expression was spotty in the peri-epithelial UM irrespective of the genotype. However, increased expression in the epithelial compartment of the *Ctnnb1-cKO* ureter was reliably found for *Sp5*, *Lix1*,* Lhx1*, *Emx2*,* Pax8* and *Hnf1b* (Fig. 2D).

The set of down-regulated genes encoded 29 TFs, for which GO terms for biological processes related to “smooth muscle differentiation” and ”epithelial differentiation” were enriched (Fig. 2E; Table S5). Some of these TF genes have previously been implicated in urothelial stratification and differentiation of B and I cells (*Trp63*) [14, 31], the differentiation of S cells (*Pparg*,* Foxa1*,* Grhl3*) [14, 25, 32–35], and SMCs (*Myocd*,* Tbx18*, *Sox9*,* Foxf1*,* Gata6*,* Gata2*,* Zeb2*) in the ureter and/or bladder [13, 17, 36–40] (Fig. 2F, Table S6).

RNA in situ hybridization analysis detected expression of *Pou3f1*,* Myocd*,* Ahr*, *Tbx18*, *Hopx*, *Lef1*,* Dach1*, *Klf15*,* Sox9*, *Zeb2*,* Tcf12*, *Foxf1*, *Zfp536*,* Gata6*, *Trps1*,* Gata2*, and *Snai1* in the mesenchymal compartment of E14.5 control ureters; expression of *Id4* also occurred in the UE. Expression of all these genes was lost in *Ctnnb1-cKO* ureters. Expression of *Trp63*, *Pparg*, *Foxq1*, *Grhl3*,* Foxa1*, and *Msx2* was confined to the UE in the control and was lost (*Trp63*, *Pparg*, *Grhl3*,* Msx2*) or downregulated (*Foxq1*,* Foxa1*) in this region in the *Ctnnb1-cKO* ureter (Fig. 2G). We conclude that mesenchymal WNT signaling is required for repression of some early UB/kidney-specific TF genes in the UE, and for activation of a large set of TF genes both in the UM and in the UE, of which some are known to mediate tissue-specific differentiation programs in the ureter.

### Loss of mesenchymal WNT signaling affects the expression of various signaling components in the early ureter

Although it is conceivable that mesenchymal WNT signaling directly activates expression of some of the pro-differentiation TF genes in the UM, (at least) the expression of TFs in the UE must be controlled indirectly by controlling expression of signals and/or signaling activities that act onto and/or in the adjacent UE. To identify such signals and signaling activities, respectively, we analyzed the set of deregulated genes in the E14.5 *Ctnnb1-cKO* ureters by KEGG pathway analysis (subprogram “KEGG2026” within the web-based “Enrichr” gene list enrichment analysis tool (https://maayanlab.cloud/Enrichr/) [26–28].

**Fig. 3:**
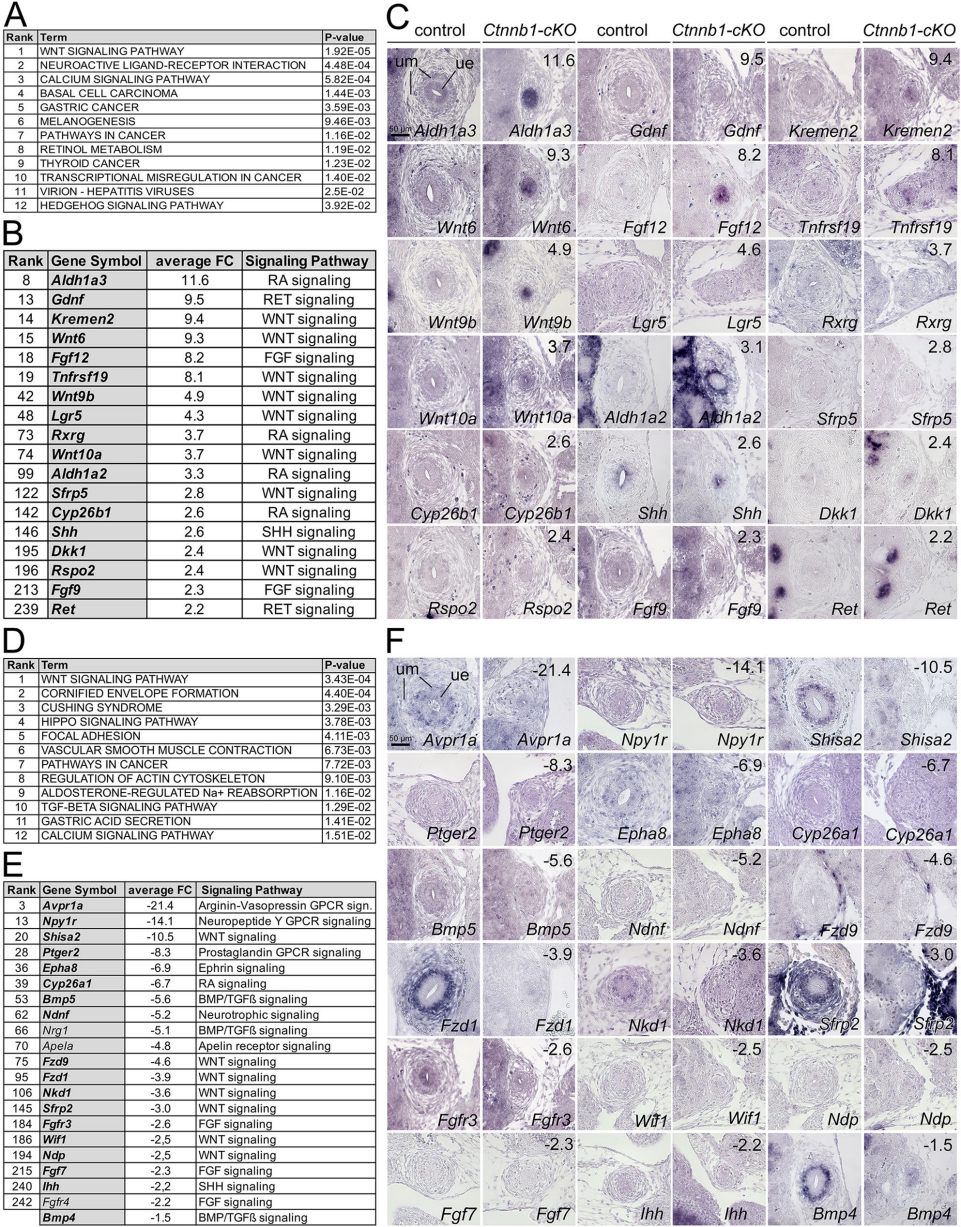
Expression of various signals and signaling components is changed in E14.5 *Ctnnb1-cKO* ureters. **A-F** Analysis of E14.5 *Ctnnb1-cKO* ureter microarrays for genes encoding signals or signaling component with increased (**A-C**) and reduced expression (**D-F**). **A**,** D** Gene ontology (GO) enrichment analysis for genes with increased expression (**A**) and for genes with reduced expression (**D**) in the E14.5 *Ctnnb1-cKO* microarray. The top 12 scoring terms are listed with their *P*-value. **B**, **E** List of genes encoding signals or signaling components with increased expression (**B**) and with reduced expression (**E**) in the E14.5 *Ctnnb1-cKO* ureter microarrays. Shown are the overall rank, the gene name, the average fold change (avgFC) of expression and the related signaling pathway. **C**,** F** RNA in situ hybridization analysis on proximal ureter sections of E14.5 control and *Ctnnb1-cKO* embryos for expression of genes encoding signals or signaling components with increased expression in microarrays of E14.5 *Ctnnb1-cKO* ureters (marked in bold in **B**) and with reduced expression (marked in bold in **E**). Numbers in the upper right corner relate to the avgFC in the microarray. *n* = 3 per probe and genotype. ue, ureteric epithelium; um, ureteric mesenchyme

For the group of upregulated genes, the term “WNT signaling pathway” was most highly enriched, underlaid by increased expression of genes encoding WNT ligands (*Wnt6*, *Wnt10a*,* Wnt9b*), WNT coactivators (*Rspo1*, *Rspo2*, *Rspo3)*, WNT antagonists (*Kremen2*, *Tnfrsf19*, *Sfrp5*,* Dkk1)*, and a WNT coreceptor (*Lgr5*). “Neuroactive ligand-receptor interaction and Calcium signaling” included amongst others the *Gdnf-Ret* ligand receptor gene pair. The term “retinol metabolism” related to increased expression of the RA-biosynthetic enzyme genes *Aldh1a1*,* Aldh1a2* and *Aldh1a3;* “Hedgehog signaling pathway” to increased expression of *Shh* (Fig. 3A, Table S7). Additional genes encoding signals with increased expression were *Fgf12*,* Fgf9* and *Fgf5* (see Fig. 3B and Table S8 for all signaling components with increased expression). We validated expression of the 10 top-upregulated signaling component genes as well as additional components of the primarily affected pathways (WNT, RA, RET-GDNF, SHH, FGF) from the list by RNA in situ hybridization analysis. We found strongly increased expression of *Aldh1a3*, *Wnt6*,* Fgf12*, *Wnt9b* in the UE, and of *Aldh1a2* in the UM of E14.5 *Ctnnb1-cKO* ureters. Expression of *Kremen2*, *Wnt10a*, and *Shh* appeared weakly increased in the mutant UE. Expression of all other components was not reliably detected (Fig. 3C).

In the set of 255 down-regulated genes, KEGG2026-based pathway analysis revealed a strong enrichment of components related to WNT signaling (including *Shisa2*,* Fzd1*, *Fzd9*,* Nkd1*, *Lef1*), Hippo signaling (which however, turned out to be mostly WNT and BMP signaling components), BMP/TGFß signaling (*Id4*,* Fmod*, *Ltbp1*,* Bmp5*, *Bmp6)* and various types of GPCR signaling mostly related to neurons (Fig. 3, D and E; Table S9 and S10). We again validated expression of the 10 top-downregulated genes encoding signaling components as well as additional components of the primarily affected pathways (WNT, FGF, BMP) by RNA in situ hybridization analysis. In addition to *Lef1* and *Tcf12* (Fig. 2G), we found reduced expression of *Avpr1a*, *Shisa2*, *Epha8*, *Bmp5*, *Fzd1*, *Nkd1* and *Sfrp2* in the UM of E14.5 *Ctnnb1-cKO* ureters. Expression of *Fgfr3* was reduced in the UE. Notably, we found strongly reduced expression of *Bmp4* in the UM, and an increased expression of this gene in the UE, which may explain the weak overall 1.5-fold downregulation in the E14.5 *Ctnnb1-cKO* ureter microarray (Fig. 3F).

Together, these findings show that loss of mesenchymal *Ctnnb1* affects the expression of signals and signaling components in the early ureter in a complicated manner. While genes encoding ligands for WNT, SHH, FGF and BMP4 signaling exhibit increased expression in the UE, signaling components for WNT and BMP4 signaling are reduced in the UM.

### Loss of mesenchymal WNT signaling leads to increased epithelial WNT signaling and decreased BMP4, SHH, FGF and RA signaling in the early ureter

We next wished to determine the consequences of the altered expression of signaling components on the activity of the respective pathways in the *Ctnnb1-cKO* ureter. We focused on WNT, FGF, SHH, BMP4 and RA signaling, since their signaling components were most reliably affected, and they are relevant for cyto-differentiation in the murine ureter [12, 13, 15, 18–20]. As read-outs, we used expression of genes, which have been recognized as *bona-fide* transcriptional targets of the respective pathway. Importantly, we screened for expression of these genes on three consecutive stages (E12.5 to E14.5) to distinguish primary from secondary changes.

**Fig. 4:**
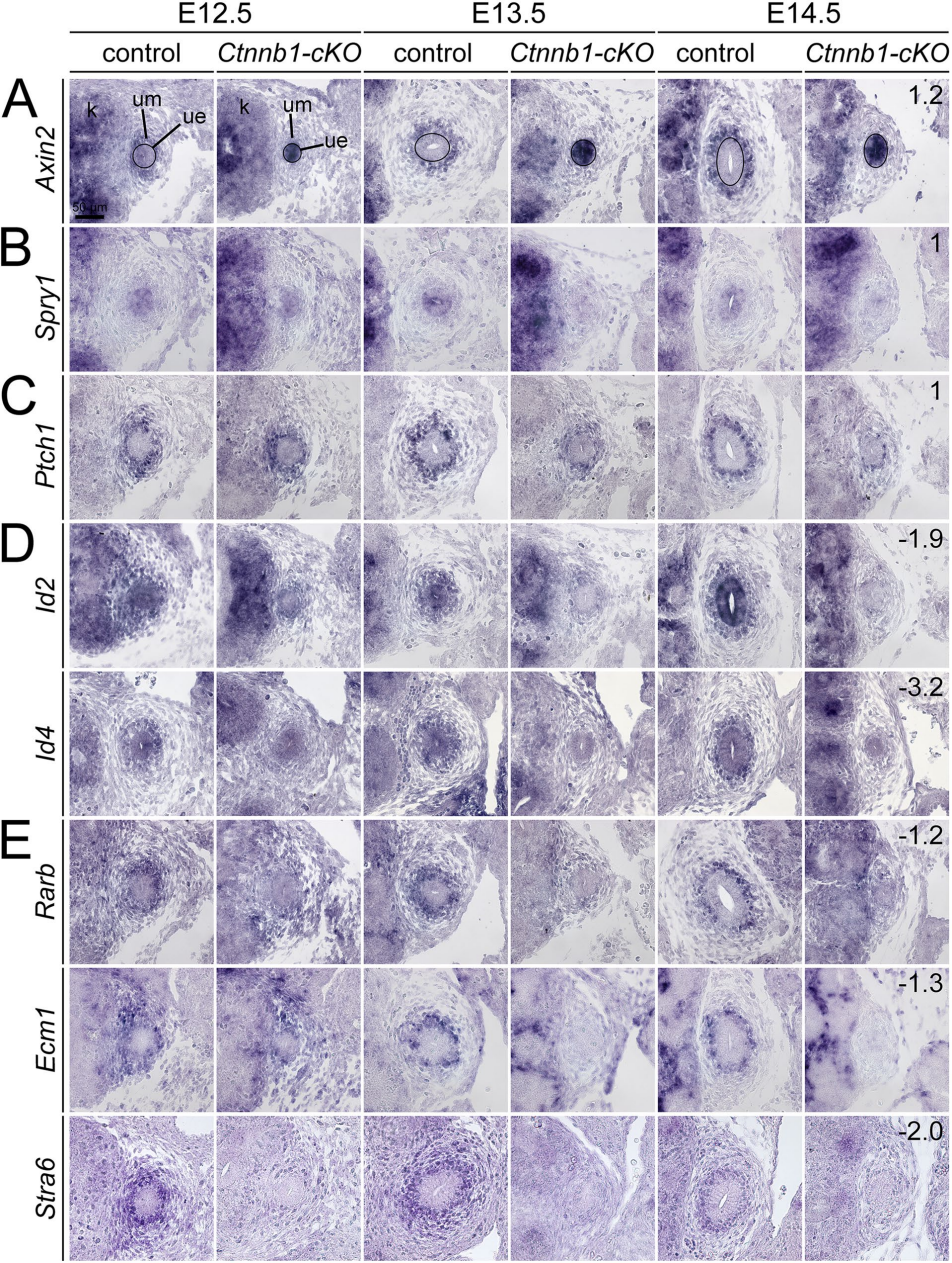
The activity of several signaling pathways is changed in *Ctnnb1-cKO* ureters. **A-E** RNA in situ hybridization analysis on sections of the proximal ureter and the attached kidney of E12.5, E13.5 and E14.5 control and *Ctnnb1-cKO* embryos for expression of genes encoding direct target genes of WNT signaling (**A**), FGF signaling (**B**), SHH signaling (**C**), BMP4 signaling (**D**), and RA signaling (**E**). *n* = 3 per probe, stage and genotype. Numbers in the upper right corner relate to the average fold change in the E14.5 *Ctnnb1-cKO* microarray. To better appreciate the shift of *Axin2* expression from the mesenchymal to the epithelial compartment of the ureter, the ureteric epithelium is demarcated by a black oval line in all images relating to this analysis (**A**). k, kidney; ue, ureteric epithelium; um, ureteric mesenchyme

With respect to WNT signaling, we found that the expression of the target gene *Axin2* [41] was confined to the UM in control embryos. In the mutant ureter, mesenchymal expression of *Axin2* was absent but strong ectopic expression in the epithelium was detected at all stages (Fig. 4A). Ectopic expression in the E14.5 UE was also found for *Sp5* (Fig. 2D), a direct target of WNT signaling in stem and progenitor cells [42, 43]. The FGF signaling target *Spry1* [44] was expressed at low levels in the UE of control embryos. In the mutant ureter, epithelial expression appeared reduced at E13.5 and E14.5 (Fig. 4B). Expression of the SHH signaling target gene *Ptch1* [45] was unchanged in the UM at E12.5, but appeared reduced at E13.5 and E14.5 (Fig. 4C).

Expression of *Id2* and *Id4*, direct targets of BMP4 signaling [46, 47], was progressively reduced both in the UM and the UE of mutant embryos from E12.5 to E14.5 (Fig. 4D). Although expression of genes encoding RA-biosynthetic enzymes was strongly increased in the UE (*Aldh1a1*, *Aldh1a3*) and the UM (*Aldh1a2*) of *Ctnnb1-cKO* embryos from E12.5 to E14.5 (Fig. S3A), expression of *Rarb*, encoding a receptor and direct target of RA signaling [48], of the RA-dependent gene in the UM, *Ecm1* [18], and the RA-responsive gene *Stra6* [49] was strongly reduced in the mutant UM (Fig. 4E). The expression of other RA-responsive genes (*Crabp2*, *Cyp26a1*, *Cyp26b1*, *Cyp26c1*, *Tgm5*) was not detected in control and mutant ureters at the analyzed stages (Fig. S3B).

The expression changes detected in the microarrays of E14.5 *Ctnnb1-cKO* ureters were consistent with most of these observations, including the fact that the overall level of *Axin2* was unchanged due to opposing changes of expression in the UE and UM (Fig. 4A-E). For some genes (*Spry1*, *Ptch1*,* Rarb* and *Ecm1*) the downregulation in the microarray of E14.5 *Ctnnb1-cKO* ureters appeared weak compared to the signaling changes identified by the RNA in situ hybridization analysis. However, microarrays average expression intensities over the entire length of the ureter and its tissues, and may be affected by the tissue hypoplasia observed in the mutant ureters.

We conclude that mesenchymal WNT signaling is required to repress epithelial WNT signaling and maintain FGF, SHH, BMP4 and RA signaling in the early ureter. Owing to the early loss of RA signaling in the UM, of BMP4 signaling both in the UM and UE, and the early onset of ectopic WNT signaling in the UE, these three pathways may present primary effectors of the epithelial differentiation defects in the *Ctnnb1-cKO* ureter.

### Ectopic epithelial WNT signaling disrupts epithelial stratification and differentiation in the ureter

**Fig. 5:**
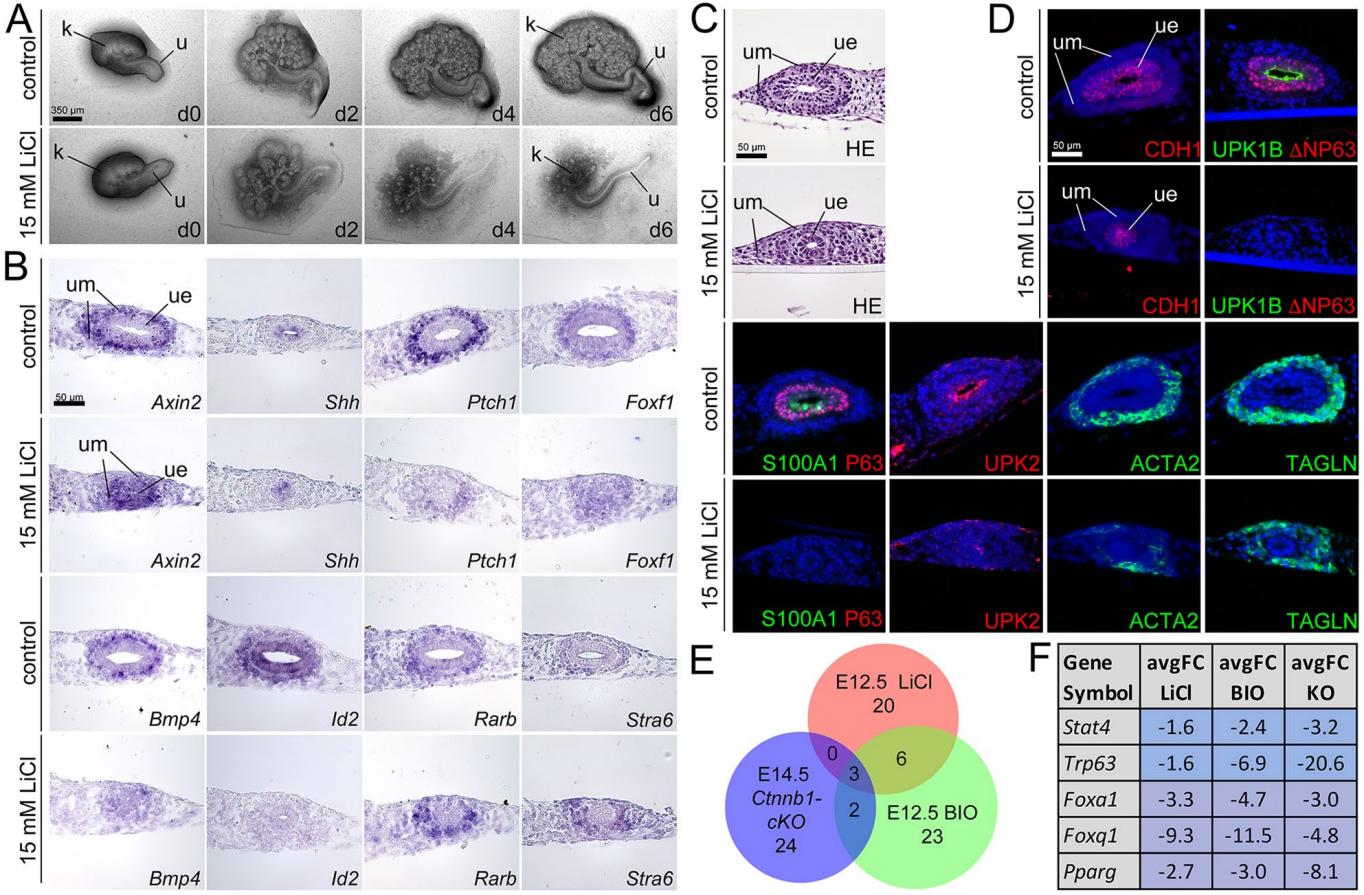
Activation of canonical WNT signaling in the UE prevents urothelial stratification and differentiation. Wildtype ureters were isolated at E12.5, and cultured for 6 days in absence (control) or presence of 15 mM of the WNT signaling agonist LiCl. **A** Morphology of kidney/ureter explants over the culture period. **B** RNA in situ hybridization analysis on transverse sections of the proximal ureter after 2 days of culture for expression of genes encoding signals and signaling targets. **C**,** D** Histological analysis by hematoxylin and eosin staining (**C**) and immunofluorescence analysis of cytodifferentiation markers (**D**) on transverse section of the proximal ureter after 6 days of culture. *n* = 5 for each assay. k, kidney; u, ureter; ue, ureteric epithelium; um, ureteric mesenchyme. **E** Overlap of transcription factor (TF) genes with reduced expression in microarrays of E12.5 ureters treated for 18 h with 15 mM LiCl (E12.5 LiCl) or 10 µM BIO (E12.5 BIO) and E14.5 *Ctnnb1-cKO* ureters. **F** List of TF genes from overlap in (**E**) with their average fold (avgFC) change of expression in microarrays of E12.5 ureters treated for 18 h with 15 mM LiCl (LiCl) or 10 µM BIO (BIO) and E14.5 *Ctnnb1-cKO* ureters (KO). Background colors relate to the colors used in (**E**)

While loss of mesenchymal *Bmp4* impairs epithelial stratification and differentiation in the developing ureter [15], and loss of RA signaling leads to premature differentiation [18], it is not known what phenotypic consequences result from ectopic WNT signaling in the UE. To address this question, we used a pharmacological approach in ureter explant cultures. For this, we cultured combined kidney/ureter explants from E12.5 wildtype embryos for 6 days in presence of 15 mM of the GSK-inhibitor LiCl [50], a concentration that has previously been shown to efficiently activate *Ctnnb1*-dependent WNT signaling in the kidney [51, 52]. After 2 days of the culture, ectopic nephrogenesis was apparent in the kidney, whereas the ureter was thinner. At the endpoint of culture at day 6, kidney development was disrupted, confirming previous reports [53]. The ureter had a normal length but remained thin (Fig. 5A).

RNA in situ hybridization analysis of 2-day ureter cultures revealed ectopic expression of the direct target gene of WNT signaling, *Axin2*, in the UE, confirming the suitability of our approach. Interestingly, the mesenchymal expression of *Axin2* appeared largely unchanged both with respect to level and localization in the inner region of the UM, indicating that the pathway cannot be enhanced or ectopically activated in the UM. Expression of *Shh* in the UE was unchanged, whereas expression of the SHH target gene *Ptch1* in the UM appeared reduced. Expression of *Foxf1*, *Bmp4* and *Id2* in the UM was also reduced, whereas expression of *Id2* in the UE was lost (Fig. 5B). Expression of the RA signaling target genes *Rarb* and *Stra6* appeared slightly increased in the UM, in line with increased expression of *Aldh1a2* in the UM and *Aldh1a3* in the UE (Fig. 5B, Fig. S4A). Expression of other RA responsive genes was not detected in either genotype (Fig. S4B). We conclude that ectopic epithelial WNT signaling weakens the SHH-FOXF1-BMP4 pro-differentiation signaling activities and increases RA signaling in the early ureter.

Analysis at the culture end-point revealed severe histological and molecular defects in LiCl-treated ureters. The epithelial compartment was mono-layered and lacked expression of markers for B, I and S cells. (Please note that we did not check expression of KRT5, since B cell differentiation is delayed in culture). SMC differentiation was weakly affected (Fig. 5, C and D). We conclude that activation of WNT signaling in the UE affects mesenchymal differentiation weakly, but disrupts urothelial stratification and differentiation.

To characterize whether ectopic WNT signaling compromises the expression of pro-differentiation TF genes in the ureter, we treated E12.5 ureter explants for a short 18 h culture period with WNT signaling activators, namely the GSK3B inhibitors LiCl (15 mM) and BIO (10 µM), respectively, and performed global transcriptional profiling of the ureter explants at the culture end-point [50, 54] These treatments affected the expression of only 5 of the 24 TF genes, which showed reduced expression in E14.5 *Ctnnb1-cKO* ureters, including *Trp63*, the regulator of epithelial stratification and I- and B-cell differentiation [14], and *Foxa1* and *Pparg*, which have been implicated in S-cell differentiation in the ureter [25, 55] (Fig. 5, E and F; Table S11-S15).

### Combined restoration of BMP4 and inhibition of epithelial WNT signaling partially improves the epithelial stratification and differentiation defects in the *Ctnnb1-cKO* ureter

**Fig. 6:**
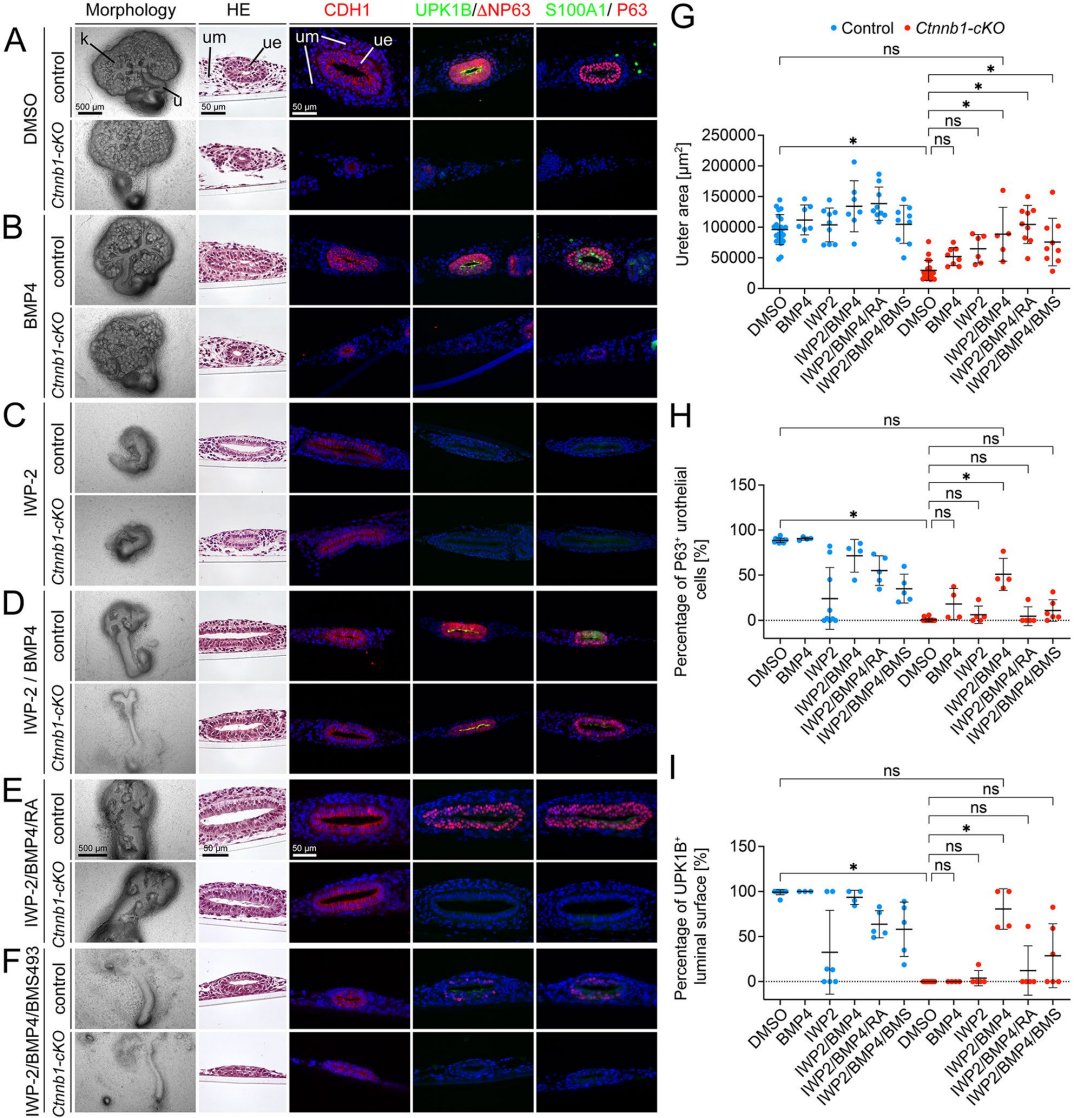
Epithelial differentiation in *Ctnnb1-cKO* ureters is partially improved by co-treatment with BMP4 and IWP-2. Control and *Ctnnb1-cKO* ureters were isolated at E11.5, and cultured for 6 days in presence of DMSO (**A**), 100 ng/ml BMP4 (**B**), 5 µM IWP-2 (**C**), 100 ng/ml BMP4 and 5 µM IWP-2 (**D**), 100 ng/ml BMP4, 5 µM IWP-2 and 1 µM retinoic acid (**E**), and 100 ng/ml BMP4, 5 µM IWP-2 and 1 µM BMS493 (**F**). Shown is the morphology of the explants after 6 days (first column), the histological staining (HE) of transverse ureter sections (second column), and immunofluorescence analysis of expression of markers for epithelial stratification (CDH1) and differentiation (S cells: UPK1B, S100A1; B and I cells: ΔNP63, P63; third to fifth column). *n* = 5 for each assay. k, kidney; u, ureter; ue, ureteric epithelium; um, ureteric mesenchyme. **G-I** Quantification of the ureter area (**G**), the percentage of P63^+^ cells (**H**) and the UPK1B^+^ domain (**I**) in ureter cultures shown in (**A-F**), *n* ≥ 4. Statistical data are presented as mean±standard deviation. Statistical significance was determined by a Kruskal-Wallis test followed by the Benjamini-Krieger-Yekutieli two-stage linear step-up procedure for multiple comparisons (FDR = 5%). Asterisks (*) indicate a discovery (*q* < 0.05); ns, not significant. See Table S16-S18 for source data and statistics

To determine whether and how the loss of BMP4 signaling, ectopic epithelial WNT signaling and altered RA signaling contribute to the urothelial defects in the *Ctnnb1-cKO* ureter, we performed pharmacological rescue experiments in cultured ureter explants. For this, we explanted the control and mutant ureters at E11.5, and cultured them for 6 days with BMP4, the WNT signaling antagonist IWP-2 [56], RA, the pan RA-receptor inhibitor BMS493 [57] and combinations thereof. At the culture end-point, we analyzed morphology, histology and expression of urothelial differentiation markers qualitatively (Fig. 6A-F), and quantified ureter size (area), the percentage of P63^+^ urothelial cells and the UPK1B^+^ luminal surface as read-outs for growth, stratification and S-cell differentiation, respectively (Fig. 6G-I, Table S16-S18).

*Ctnnb1-cKO* ureters were hypoplastic and showed a complete lack of epithelial stratification and differentiation in culture (Fig. 6, A and G-I). Treatment with 100 ng/ml BMP4, a concentration previously shown to elicit robust BMP4 signaling in ureter explant cultures [11], did not affect the ureter area and urothelial stratification and differentiation in control ureters, and did not rescue the histological and molecular defects in the mutant ureter (Fig. 6, B and G-I). Treatment of control and *Ctnnb1*-deficient ureters with 5 µM of the WNT antagonist IWP-2, a concentration previously shown to efficiently inhibit canonical WNT signaling in cultured ureters [11], led to an arrest of kidney development and to a short but broadened ureter, which completely lacked urothelial stratification and differentiation (Fig. 6, C and G-I). Both control and *Ctnnb1-cKO* ureters treated with a combination of BMP4 and IWP-2 elongated over the culture period and appeared wider in circumference. They had a two-layered epithelial compartment and expressed ΔNP63/P63 in the basal layer and weakly UPK1B and S100A1 in the luminal layer (Fig. 6D). Quantification revealed a significant increase of the ureter area, P63 expressing cells and the UPK1B^+^ domain in *Ctnnb1-cKO* ureters co-treated with BMP4 and IWP-2 compared to untreated mutant ureters (Fig. 6G-I). This suggests that both loss of BMP4 signaling and gain of epithelial WNT signaling cause or at least contribute to the epithelial stratification and differentiation defects in *Ctnnb1-cKO* ureters.

Since RA production and RA signaling were inversely affected in *Ctnnb1-cKO* ureters, we addressed a possible contribution of both increased and decreased RA signaling to the cellular defects in mutant ureters. Addition of 1 µM RA or of 1 µM BMS493 to explant cultures of *Ctnnb1-cKO* ureters co-treated with BMP4 and IWP-2 increased the ureter area compared to the untreated mutant ureter, but did not increase the expression of P63 and of S-cell markers compared to the untreated mutant ureter (Fig. 6E -I), suggesting that reduced RA signaling contributes to the growth deficit but not to the cyto-differentiation defects in the *Ctnnb1-cKO* ureter.

### Cyto-differentiation of the ureter requires WNT signaling in the early undifferentiated UM

To identify direct targets of WNT signaling in the UM, we deemed a short-term pharmacological WNT inhibition in ureteral explant cultures a suitable approach. In such a setting, epithelial WNT signaling is suppressed, and only genes, which directly or exclusively depend on WNT signaling in the UM should be strongly attenuated in their expression. To define which ureter stage is best suited for this approach, we explanted ureters at E11.5, E12.5, E14.5 and (E18.5 as a control) and cultured them with 5 µM IWP-2, a concentration previously found to strongly inhibit WNT signaling in kidney cultures, for different times to reach a stage similar to early postnatal development. We monitored the morphology and performed histological and marker analysis for SMC differentiation at the culture end-point and quantified ureter size (area) and ACTA2^+^ SMCs (Fig. S5, Table S19 and S20).

Ureters explanted at E11.5 or E12.5 and cultured for 11 or 10 days, respectively, in presence of IWP-2 remained shorter but increased in diameter, resulting in an overall unchanged ureter area; they showed (strongly) reduced SMC investment compared to the control (Fig. S5,A, B, E, I; Table S19 and S20). Ureters explanted at E14.5 and E18.5 and cultured with IWP-2 for 8 or 4 days, respectively, had a normal size and unchanged SMC investment, but expression of the late SMC marker CKM appeared reduced (Fig. S5C-F, Table S19 and S20). We conclude that mesenchymal WNT signaling is most strongly required for SMC development in the early undifferentiated ureter at E11.5 and E12.5. Interestingly, no statistically significant epithelial differentiation defects were found in E11.5 or later explants treated with 5 µM IWP-2 (Fig. S6, Table S21, also compare Fig. S6 to Fig. 6A) suggesting that ectopic epithelial WNT signaling, that cannot occur upon IWP-2 treatment, is the major driver of epithelial defects in the *Ctnnb1-cKO* ureter.

### Mesenchymal WNT signaling affects the SHH-FOXF1-BMP4 signaling axis

**Fig. 7:**
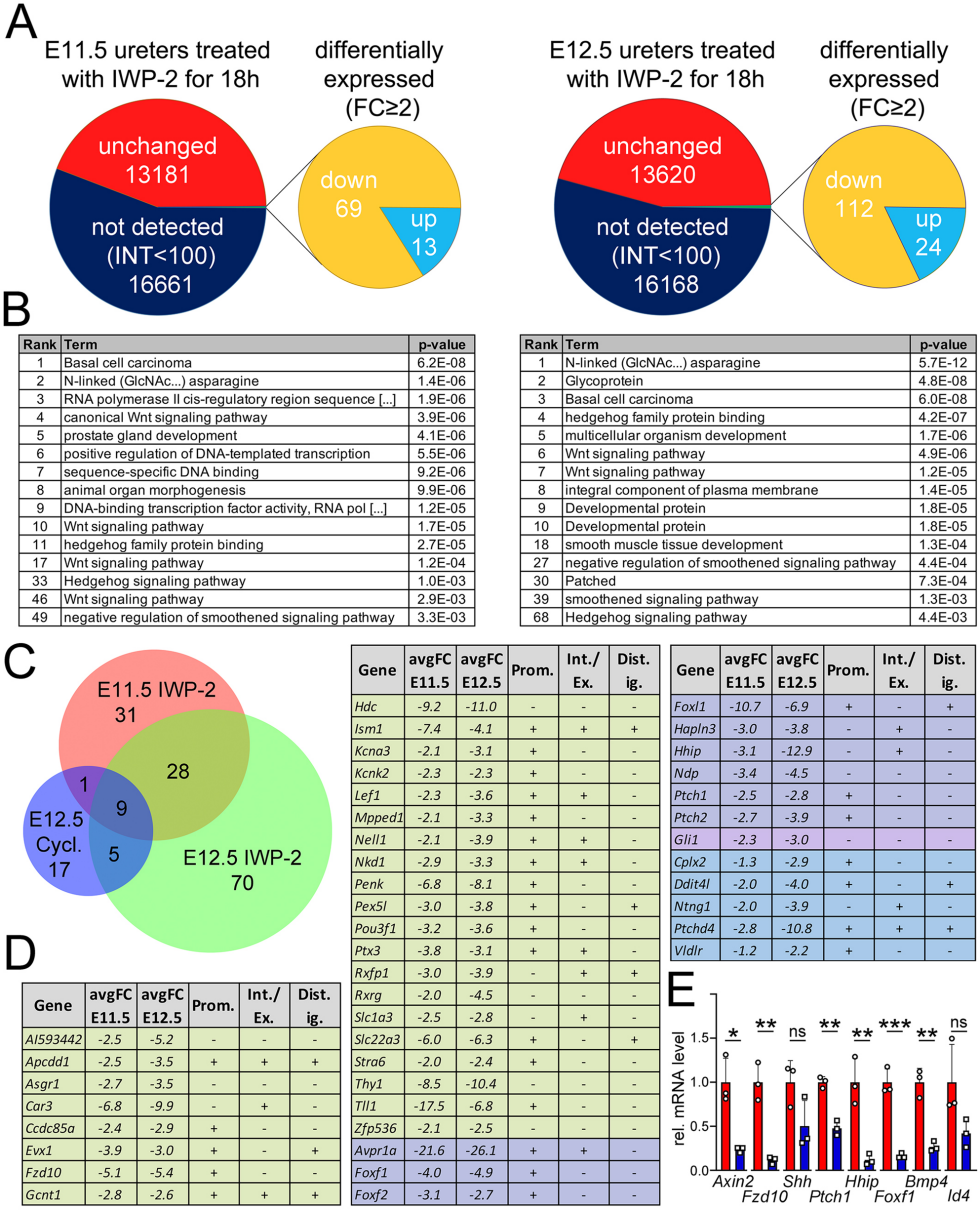
The SHH-FOXF1-BMP4 axis is a target of canonical WNT signaling in the early UM. **A**,** B** Wildtype ureters were isolated at E11.5 and E12.5 and cultured for 18 h with 10 µM of the WNT signaling inhibitor IWP-2 before they were subjected to global transcriptome analysis. **A** Pie chart summarizing the results of the microarray analysis of two pools each of E11.5 and E12.5 ureters, untreated or treated with IWP-2. **B** Gene ontology (GO) enrichment analysis using DAVID for downregulated genes in microarrays of ureter explants from E11.5 (left) and E12.5 (right) ureters. The top 10 scoring terms as well as selected others are listed with their *P*-value. **C** Venn diagram showing the overlap of gene sets with reduced expressed in E11.5 (red) and E12.5 ureters (green) treated with 10 µM IWP-2 for 18 h, and in E12.5 ureters treated with 10 µM cyclopamine for 18 h (blue). **D** List of genes with reduced expression in 11.5 and/or E12.5 ureters treated with 10 µM IWP-2 for 18 h, and with/without reduced expression in E12.5 ureters treated with 10 µM cyclopamine for 18 h. Background colors relate to colors used in 7** C.** For each gene the average fold change (avgFC) in the E11.5 + 18 h and E12.5 + 18 h microarray is shown as well as the presence of LEF/TCF and CTNNB1 binding sites in promoter (prom.), intron/exon (in./ex.) or distal intergenic regions (Dist. ig.). See Table S32 for a complete list of CTNNB1 peaks in differentially expressed genes. **E** RT-qPCR analysis for selected candidate genes in E12.5 ureters treated with 10 µM IWP-2 for 18 h. *n* = 3. Statistical data are presented as mean±standard deviation. Significant differences (unpaired Student’s t-test) are indicated (*P < 0.05; **P < 0.01; ***P < 0.001). See Table S30 for RT-qPCR source data and statistics

Due to the above results, we explanted wild-type ureters at E11.5 and E12.5, cultured them for a short time (18 h) in the presence of a high dose of IWP-2 (10 µM) and profiled the global transcriptional changes. Using intensities > 100 and fold changes (FCs) > 2 as cut-offs for robust transcriptional changes in the two individual microarrays performed for each stage, we identified 13 upregulated and 69 downregulated genes for E11.5 explants and 24 upregulated and 112 down-regulated genes for E12.5 explants (Fig. 7A, Tables S22-S25). For the set of down-regulated genes at E11.5 and E12.5, the DAVID software tool [58] identified enrichment of genes associated with “WNT signaling pathway” and “smooth muscle tissue development” in agreement with a successful inhibition of mesenchymal WNT signaling (and absence of ectopic epithelial activation of the pathway) and a primary activity in SMC differentiation. Interestingly, we also found the GO terms “basal cell carcinoma” and “hedgehog family and signaling” in both gene lists (Fig. 7B, Tables S26, S27). Intersecting the lists of downregulated genes at E11.5 and E12.5 identified 37 genes that were in common (Fig. 7, C and D; Table S28). An overlap with the list of genes downregulated by treatment of ureters with the SHH inhibitor cyclopamine at E12.5, which we previously published [13] (Table S29), uncovered that WNT signaling inhibition affects expression of almost half of the SHH-signaling dependent genes in the early ureter (Fig. 7, C and D). These genes included *Hhip*, a direct target and inhibitor of SHH signaling, the direct target and receptor gene *Ptch1*, but also members of the *Foxf1/Foxf2/Foxl1* genomic cluster which have previously been shown to mediate SHH signaling function in the early ureter [13]. Expression of *Bmp4*, which acts downstream of SHH and FOXF1 in the early ureter [13], was reduced in E12.5 ureters treated with IWP-2 (Table S24). (Strong) down-regulation of *Axin2*, *Fzd10*, *Ptch1*, *Hhip*, *Foxf1* and *Bmp4* in E12.5 ureters treated with 10 µM IWP-2 was confirmed by reverse transcription quantitative polymerase chain reaction (RT-qPCR) (Fig. 7E, Table S30).

Since the paucity of cells in the undifferentiated UM demands an excessively large number of embryos for global studies of DNA occupancy of TFs, we mined publicly available ChIP-Seq data sets of a variety of mouse tissues to identify genes likely to present direct targets of WNT signaling activity (Table S31). Specifically, we searched TF ChIP-seq datasets for binding peaks for mouse CTNNB1 in combination with at least one binding peak for the CTNNB1-binding partners TCF7, TCF7L1, TCF7L2 and LEF1 and mapped them to nearby genes. We identified common binding peaks in many of the genes associated with the GO term WNT signaling (such as *Fzd10*,* Lef1*, *Nkd1*) but also in the *Foxf1/Foxf2/Foxl1* cluster, in *Ptch1*,* Ptch2* and in *Bmp4*, suggesting that CTNNB1-dependent WNT signaling directly activates expression of multiple genes in the SHH-FOXF1-BMP4 signaling axis (Fig. 7D, Table S32).

### WNT signaling differentially affects the components of the SHH-FOXF1-BMP4 signaling axis in the UM

To discriminate how WNT signaling affects the components of the SHH-FOXF1-BMP4 signaling axis, we explanted E12.5 ureters, cultured them for 2 days in presence of 5 µM IWP-2 with and without pathway activators and scored for expression of relevant targets or effector genes by whole mount RNA in situ hybridization. As expected, expression of the WNT target gene *Axin2* was abolished by 5 µM IWP-2 and was not restored by administration of the SHH agonist purmorphamine or BMP4. IWP-2 attenuated SHH signaling in absence or presence of purmorphamine as seen by (weakly) reduced expression of *Ptch1*. Expression of the effector gene *Foxf1* was more strongly reduced, in line with an independent WNT input into activation of this gene. We also found reduced expression of the targets of BMP4 signaling *Gata6* and *Id2* in presence of IWP-2/BMP4 (Fig. S7).

**Fig. 8:**
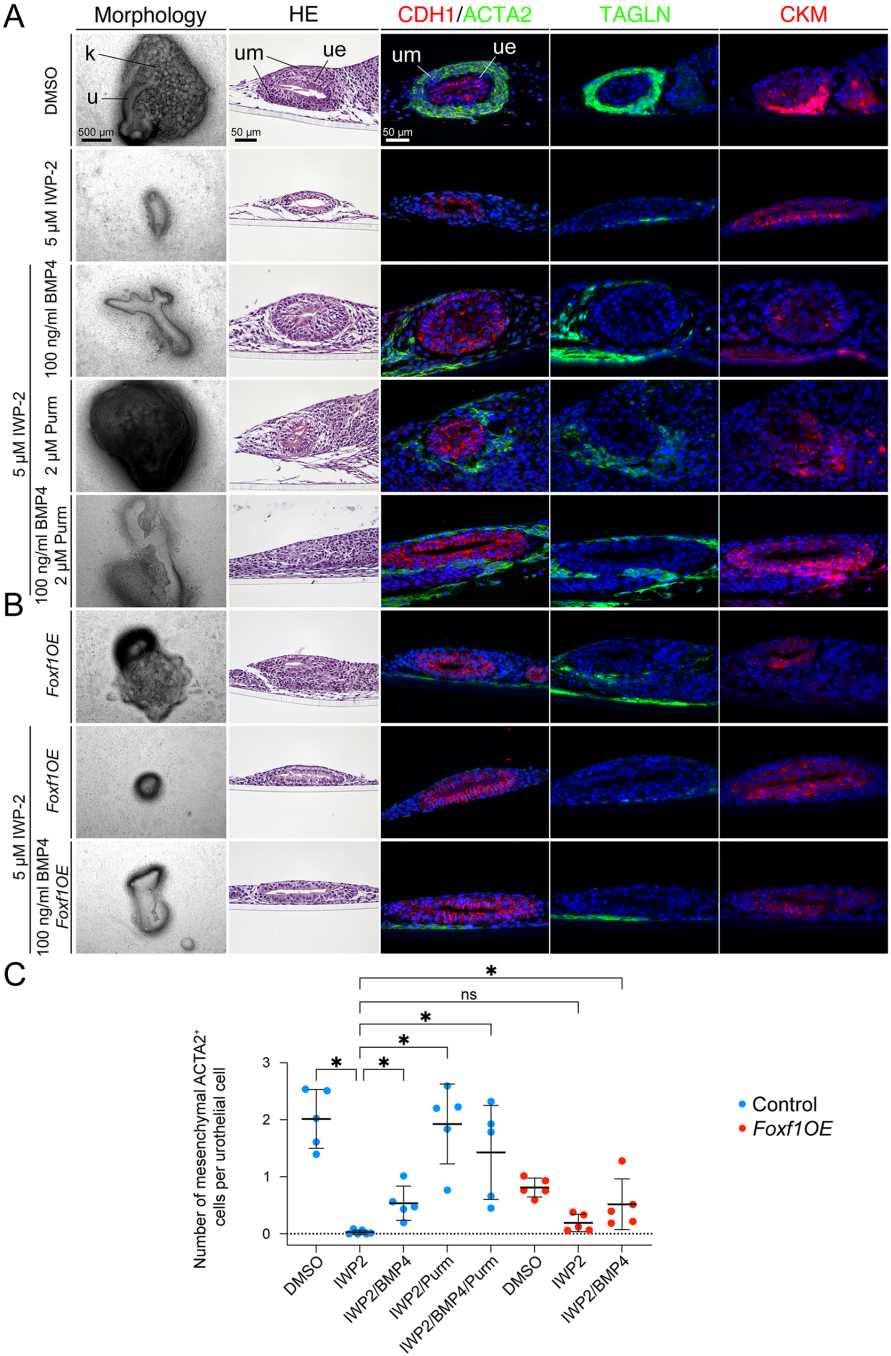
WNT signaling differentially affects the components of the SHH-FOXF1-BMP4 signaling axis in the UM. **A**,** B** Wildtype (**A**) or *Tbx18*^*creERT/+*^*Hprt*^*Foxf1*^(*Foxf1OE*) (**B**) ureters were isolated at E11.5 and cultured for 11 d with DMSO, or with 5 µM IWP-2, with or without 100 ng/ml BMP4 and/or 2 µM purmorphamine. Ureters were documented for morphology (Morphology, column 1), for histological appearance by hematoxylin and eosin staining (HE, column 2), and for expression of the epithelial marker CDH1 and the smooth muscle cell markers ACTA2, TAGLN and CKM at the end of culture period (column 3 to 5). *n* = 5 for each assay. k, kidney; nd, nephric duct; u, ureter; ue, ureteric epithelium; um, ureteric mesenchyme. **C** Quantification of ACTA2^+^ cells in the ureter cultures shown in **A** and **B**, *n* ≥ 5. Statistical data are presented as mean±standard deviation. Statistical significance was determined by a Kruskal-Wallis test followed by the Benjamini-Krieger-Yekutieli two-stage linear step-up procedure for multiple comparisons (FDR = 5%). Asterisks (*) indicate a discovery (*q* < 0.05); ns, not significant. See Table S33 for source data and statistics

To investigate the relevance of the individual components of the SHH-FOXF1-BMP4 signaling module for the loss of SMC differentiation in ureters lacking WNT signaling, we explanted ureters at E11.5, cultured them for 11 days in presence of 5 µM IWP-2 and added 100 ng/ml BMP4 and/or 2 µM of the SHH signaling agonist purmorphamine, and overexpressed *Foxf1* from a conditional misexpression allele [13] (*Tbx18*^*creERT2/+*^;*Hprt*^*Foxf1/y or +*^) in presence/absence of 100 ng/ml BMP4. Administration of BMP4 partly restored expression of SMC markers in IWP-2-treated ureters at the end of the culture period. Purmorphamine treatment, alone or in combination with BMP4, effectively increased SMC differentiation (Fig. 8, A and C; Table S33). Forced expression of *Foxf1* had no effect on SMC differentiation on its own (Fig. 8, B and C; Table S33). This suggests that WNT signaling provides positive and individual input into SHH and BMP4 signaling in the inner mesenchymal region of the early ureter.

## Discussion

### Mesenchymal WNT signaling enhances mesenchymal *Bmp4* expression and represses epithelial WNT signaling to ensure normal development of the epithelial compartment in the ureter

Our study revealed that *Ctnnb-1cKO* ureters are deficient for SMCs, as previously reported, but also exhibit a complete lack of stratification and differentiation in the epithelial compartment before birth. Although (minor) changes in adherens junctons may contribute to these defects, we believe that the primary cause is the failure to activate the expression of the transcriptional regulator of epithelial stratification and B and I cell differentiation, ΔNP63 [14], as well as of the drivers of S cell differentiation, *Pparg* and *Grhl3* [25, 35], at the end of the undifferentiated phase. Importantly, the mutant UE did not undergo a shift to a renal collecting duct fate, but rather expressed signaling components and TF genes specific for the ND and early UB, consistent with an arrest of epithelial development in *Ctnnb1-cKO* ureters at the precursor stage.

Since mice with a conditional loss of *Bmp4* in the UM also exhibit an undifferentiated epithelial compartment [15], and *Bmp4* expression and BMP4 signaling is strongly reduced in early *Ctnnb1-cKO* ureters (this study), the loss of *Bmp4* may be responsible for the epithelial defects in *Ctnnb1-cKO* ureters. However, administering BMP4 to *Ctnnb1-cKO* ureter explant cultures did not correct these defects, suggesting that altered activities of other signaling systems also contribute to the epithelial defects.

CTNNB1-dependent (canonical) WNT signaling occurs in the ND, the common primordium of the renal collecting duct system and the UE [59]. After UB formation at E10.5, WNT signaling continues in the epithelium of the distal UB - the primordium of the UE - until E11.5. However, it is then downregulated as WNT signaling increases in the surrounding UM [20].

Our study found ectopic *Axin2* and *Sp5* expression, and therefore canonical WNT signaling, in the UE of *Ctnnb1-cKO* embryos from E12.5 to E14.5, indicating that mesenchymal WNT signaling is necessary to repress epithelial WNT signaling in the early ureter. Ectopic epithelial WNT signaling was accompanied by reduced expression of *Wnt7b* in the UE and *Fzd1* in the UM. These genes encode the most likely ligand/receptor pair for mesenchymal WNT signaling [20]. In contrast, the expression of *Wnt9b* and *Wnt6* was upregulated in the UE, which is compatible with an autocrine mode of signaling triggered by the encoded ligands of these genes.

Importantly, LiCl-mediated activation of epithelial WNT signaling in wildtype ureter explant cultures resulted in a complete failure of epithelial stratification and differentiation. However, inhibiting (endogenous and ectopic) WNT signaling with IWP-2 treatment did not completely rescue the epithelial defects in *Ctnnb1-cKO* ureter cultures. Together with the partial improvement of the epithelial defects upon cotreatment of *Ctnnb1-cKO* ureters with BMP4 and IWP-2, this suggests that ectopic WNT signaling and reduced BMP4 expression contribute additively to the epithelial defects in *Ctnnb1-cKO* ureters.

Our findings add onto the results from previous studies investigating the role of WNT signaling in the ND and/or renal collecting duct system. Conditional loss of *Ctnnb1* in the ND resulted in hypoplasia/aplasia or dysplastic/cystic kidneys, which were linked to severe branching defects of the UB tips. Markers of differentiated collecting ducts were prematurely expressed in the ND/UB. Markers for ND and UB were reduced in their expression. Conversely, expression of a stabilized form of CTNNB1 appeared to block the differentiation of the collecting ducts [59, 60].

It is conceivable that, as in the ND and early UB, ectopic WNT signaling in the UE maintains the expression of TF genes related to stemness. Ectopic expression in the UE of *Ctnnb1-cKO* embryos of *Sp5*, a WNT-dependent gene enriched in stem and progenitor cells [42], supports this notion. However, it is also possible that epithelial WNT signaling represses TF genes crucial for urothelial differentiation. In fact, our transcriptional profiling of ureter explants treated with the WNT signaling activators LiCl and BIO for a short time, revealed a strong downregulation of a small set of TF genes, of which *Trp63*, *Foxa1* and *Pparg* are known to be essential for epithelial stratification and differentiation in the ureter and/or bladder [14, 25, 32, 55]. Moreover, epithelial WNT signaling may (additionally) act by inhibiting epithelial BMP4 signaling, thereby preventing the expression of pro-differentiation TF genes such as the ones mentioned above. Our finding that *Id2* expression, i.e. BMP4 signaling, is absent from the UE in wildtype ureters treated with LiCl provides evidence for this.

While the relevance of ectopic WNT signaling for the epithelial defects in *Ctnnb1-cKO* ureters is obvious, the molecular pathway by which mesenchymal WNT signaling represses epithelial WNT signaling in the normal ureter remains unclear. We have recently demonstrated that *Tbx18* plays a permissive role in ureter specification by repressing the metanephric mesenchymal gene program in the UM [37, 61]. Notably, *Axin2* expression is increased in the UE of *Tbx18-*deficient embryos at E12.5 [37], suggesting that derepression of epithelial WNT signaling is a consequence of a switch from a ureter to a kidney fate. Although *Tbx18* expression is lost in the UM of *Ctnnb1-cKO* embryos at E12.5 [20], a normal subdivision of the upper urinary tract in the ureter and kidney was observed, arguing against a fate switch from ureter to kidney in these mutants. Furthermore, the genetic restoration of *Tbx18* expression in the UM of *Ctnnb1-cKO* embryos did not suppress *Axin2* expression in the UE and failed to rescue the epithelial (and mesenchymal) cytodifferentiation defects [20], ruling out any causal involvement of *Tbx18* in the repression of epithelial WNT signaling in the ureter after E11.5.

It is likely that mesenchymal WNT signaling requires secondary signals to repress epithelial WNT signaling in the ureter after E11.5. Our expression analysis has shown that the expression of various signals including *Bmp4*,* Fgfs* and RA is affected in *Ctnnb1-cKO* ureters. As individual loss of BMP4, RA and FGF signaling has not been reported to cause ectopic epithelial WNT signaling in the ureter after E11.5 [12, 15, 18, 19], a combination of these signals, as well as yet unknown signals, may be instrumental in this regulatory program. Interestingly, we found reduced expression of *Dkk2* in the transcriptional profiling of E12.5 ureter explants treated for 18 h with IWP-2. Since *Dkk2* encodes a secreted antagonist of the WNT co-receptors RSPO [62], an involvement of DKK2 in inhibiting epithelial WNT signaling in the early ureter is possible.

### Mesenchymal WNT signaling impinges on the mesenchymal SHH-FOXF1-BMP4 signaling axis in the early ureter

Our previous work has shown that the closely related T-box transcription factor genes *Tbx2* and *Tbx3* are targets of CTNNB1-dependent WNT signaling in the UM [21]. Conditional loss of *Tbx2/Tbx3* in this region lowers the activity of two key drivers of the SMC program: *Foxf1* and BMP4 signaling, resulting in decreased SMC differentiation and increased extracellular matrix [21]. Notably, SMC differentiation in *Tbx2/Tbx3* conditional double mutants is reduced but not eliminated unlike in *Ctnnb1-cKO* ureters [20, 21]. This suggests that mesenchymal WNT signaling employs alternative effector genes to activate *Myocd* and the SMC differentiation program in the UM. Since we found that ectopic epithelial WNT signaling affects the SHH-FOXF1-BMP4 signaling axis and reduces SMC differentiation in *Ctnnb1-cKO* ureter, we used ureter explants from wildtype embryos, in which all WNT signaling (endogenous mesenchymal and ectopic epithelial) was pharmacologically inhibited for a short time in the early undifferentiated phase, to identify transcripts that depend directly on mesenchymal WNT signaling. Our unbiased profiling yielded a small set of genes, of which many contained binding sites for CTNNB1 and LEF/TCF transcription factors, proving the suitability of our approach for identifying direct target genes of canonical WNT signaling in the UM. In addition to classical WNT signaling components, we found that various components of the SHH-FOXF1-BMP4 signaling axis are likely to be direct targets of WNT signaling in the UM. These included the receptor genes *Ptch1* and *Ptch2*, the *Foxf1/Foxf2/Foxl1* cluster genes, and *Bmp4.* Our pharmacological rescue experiments confirmed that, SHH and BMP4 signaling need to be activated by WNT signaling to get robust SMC differentiation.

**Fig. 9:**
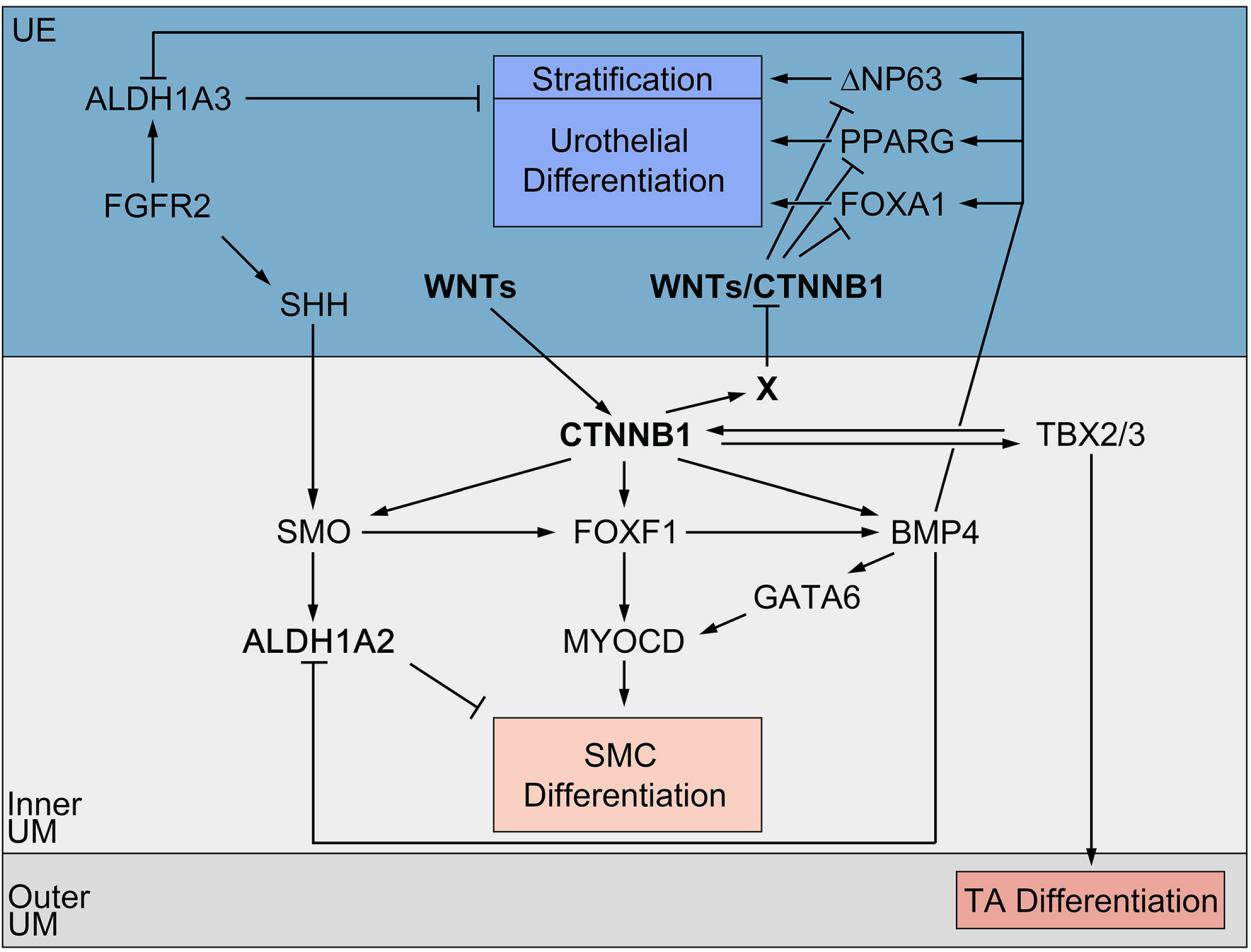
Simplified scheme of WNT signaling activity in the early ureter. Interplay of WNT, FGF, SHH, BMP, and RA signaling pathways regulating mesenchymal and epithelial differentiation in the developing ureter. Epithelial WNT signals (most likely WNT7B) enhance via the canonical (CTNNB1-dependent) subbranch the expression of components of the SHH-FOXF1-BMP4 signaling axis to increase and localize SMC differentiation to the inner region of the UM. Mesenchymal WNT signaling also activates expression of TBX2 and TBX3, which suppress the adventitial differentiation program in the inner region of the UM. Moreover, mesenchymal WNT signaling via an unknown factor X in the UM represses CTNNB1-dependent WNT signaling in the UE. By this, repression of pro-differentiation transcription factor genes *(ΔNp63*, *Pparg*, *Foxa1*) is released allowing epithelial stratification and differentiation. Components of the WNT pathway are marked in bold. Biological processes are enclosed in boxes. Arrows represent activation; bars represent repression. SMC, smooth muscle cells; TA, tunica adventitia; ue, ureteric epithelium; um, ureteric mesenchyme

Since mesenchymal WNT signaling acts in a paracrine yet very short-range fashion, enhancement of the SHH-FOXF1-BMP4 signaling axis may provide a means to confine SMC differentiation to the inner layer of the UM, and via activation of *Tbx2* and *Tbx3* restrict the adventitial fate to the outer mesenchymal cell layer in the early ureter. Together with its role in repressing epithelial WNT signaling, mesenchymal WNT signaling presents as a crucial player in the signaling network that enables the coordinated expansion and subsequent exit of the epithelial and mesenchymal tissue compartments of the early ureter from the precursor state into the differentiation programs (Fig. 9).

## Materials and methods

### Mice

The mouse alleles used were maintained on a Naval Medical Research Institute (NMRI) outbred background. This included a loss-of-function allele of *Tbx18* generated by insertion of the *cre* gene into the start codon [*Tbx18*^*tm4(cre)Akis*^, synonym: *Tbx18*^*cre*^] [63], a tamoxifen-inducible *Tbx18*^*creERT2*^ mouse line [*Tbx18*^*tm3.1(cre/ERT2)Sev*^] [64], a floxed loss-of-function line for β-Catenin [*Ctnnb1*^*tm2Kem*^, synonym: *Ctnnb1*^*fl.*^] [65] and a misexpression allele for *Foxf1* [*Hprt1*^*tm5(CAG−Foxf1,−EGFP)Akis*^, synonym: *Hprt*^*Foxf1*^] [13]. In the latter, a bicistronic transgene-cassette with a cDNA for *Foxf1* followed by a fragment harboring an IRES-GFP sequence was inversely integrated in the ubiquitously expressed X-chromosomal Hypoxanthine guanine phosphoribosyl transferase (*Hprt*) locus. After cre-mediated recombination (inversion and excision), transgene expression occurred.

Embryos were derived from matings of NMRI wild-type mice, from matings of *Tbx18*^*cre/+*^;*Ctnnb1*^*fl/+*^ males and *Ctnnb1*^*fl/fl*^ females, and from matings of *Tbx18*^*creERT2/+*^ males and *Hprt*^*Foxf1/Foxf1*^ females.

For timed pregnancies, vaginal plugs detected in the morning after mating were designated as embryonic day (E)0.5 at noon. Embryos and organ rudiments for paraffin-wax sections were dissected in PBS, fixed in 4% paraformaldehyde (PFA) in PBS and stored in methanol at -20 °C. Genomic DNA was prepared from yolk sacs or ear clip biopsies for genotyping by PCR.

Mice were housed in rooms with controlled light and temperature. The experiments were in accordance with the German Animal Welfare Legislation and approved by the local Institutional Animal Care and Research Advisory Committee and authorized by the Lower Saxony State Office for Consumer Protection and Food Safety (AZ 33.12-42502-04-13/1356, AZ42500/1H).

### Organ cultures

Ureters with or without kidneys were dissected in L-15 Leibovitz medium (#BS.F1315, Bio&SELL, Nürnberg, Germany), placed on 0.4 μm polyester membrane Transwell supports (#3450, Corning, Glendale, AZ, USA) and cultured at the air-liquid interface with DMEM/F12 (#21331020, Gibco, Waltham, MA, USA) supplemented with 10% FCS (S0115, Biochrom, Berlin, Germany), 1x penicillin/streptomycin (#15140122, Gibco), 1x nonessential amino acids (#11140035, Gibco), 1x pyruvate (#11360070, Gibco) and 1x GlutaMAX (#35050038, Gibco) in a humidified incubator with 5% CO_2_ at 37 °C. Organ cultures for microarrays and reverse transcription-quantitative PCR (RT-qPCR) were performed without FCS. Pathway activating and inhibiting compounds were added at the following final concentrations: BMP4 (100 ng/ml, #5020-BP, R&D Systems, Minneapolis, USA), IWP-2 (5 µM in DMSO for long term cultures, 10 µM for 18 h cultures; #S7085, Selleck Chemicals, Houston, USA), LiCl (15 mM in water, Carl Roth, Karlsruhe, Germany), purmorphamine (2 µM in DMSO, #S3042, Selleck Chemicals, Houston, USA), BIO (10 µM in DMSO, #S7198, Selleck Chemicals), RA (1 µM in DMSO, #0695, Tocris Bioscience, Bristol, UK) and BMS493 (1 µM in DMSO, #3509, Tocris Bioscience). The medium containing components or their solvents was refreshed every other day. In order to induce recombination in *Tbx18*^*creERT2/+*^;*Hprt*^*Foxf1/+*^ ureter explant cultures, 4-hydroxytamoxifen (H7904, Sigma-Aldrich, St. Louis, USA) was added to the medium at a final concentration of 500 nM for the first 48 h.

### Immunofluorescence analysis

Immunofluorescence staining was performed on 5-µm paraffin wax sections. Labelling with primary antibodies was performed at 4 °C overnight after antigen retrieval (15 min at 100 °C; H-3300, Vector Laboratories, Newark, CA, USA), blocking of endogenous peroxidases with 3% H_2_O_2_/PBS for 15 min and incubation in blocking buffer (TNB) provided by the Tyramide Signal Amplification (TSA) kit (NEL702001KT, Akoya Biosciences, Marlborough, MA, USA) for 45 min. Bound primary antibodies were detected with fluorophore-labelled secondary antibodies. In case signal amplification was required, we used biotinylated secondary antibodies in combination with the TSA kit. The latter contained a fluorescently labeled streptavidin conjugate to yield a largely increased fluorescent signal intensity. Nuclei were counterstained with 4′,6-diamidino-2-phenylindole (DAPI, 0.5 µg/ml, 6335.1, Carl Roth, Karlsruhe, Germany). A list of all antibodies used in this study can be found in Table S34.

### RNA in situ hybridization analysis

RNA in situ hybridization analysis was performed on 10-µm paraffin sections or on whole kidney explants using digoxigenin-labeled antisense riboprobes as previously described [66, 67].

### Quantification of growth and differentiation in ureter explant cultures

For the quantitative evaluation of cultured ureters, brightfield and immunofluorescence images were analyzed using FIJI (version 2.16.0/1.54p) and Adobe Photoshop CC (Adobe, San Jose, CA, USA). Overall morphometric measurements of the cultured organs were performed on brightfield overview images by tracing the outer lining with the Polygon Selection tool in FIJI to determine the total ureter area (µm^2^). Cell counting was conducted manually in Adobe Photoshop CC using the Count Tool. On ACTA2 and CDH1 co-stained immunofluorescence sections, ACTA2^+^ SMCs were counted and normalized to the number of epithelial cells (CDH1^+^/DAPI^+^) to calculate the SMC-to-urothelial cell ratio. P63^+^ and S100A1^+^ urothelial cells were quantified on immunofluorescence sections and expressed as the percentage of P63^+^ or S100A1^+^, respectively, cells relative to total DAPI^+^ urothelial nuclei.

To assess S-cell differentiation, the luminal perimeter of UPK1B-stained sections was determined by tracing the luminal border using the Polygon Selection tool in FIJI. Concurrently, individual UPK1B-positive segments along the luminal membrane were measured using the Segmented Line tool. The cumulative length of these positive patches was calculated in Microsoft Excel (MicrosoftCorp, Redmond, WA, USA) and divided by the total luminal perimeter to determine the percentage of UPK1B-positive luminal surface.

Statistical analysis was performed using GraphPad Prism (version 7a). Data groups were compared using a non-parametric Kruskal-Wallis test. To correct for multiple comparisons among the pre-defined pairs, the two-stage linear step-up procedure of Benjamini, Krieger, and Yekutieli was applied. The False Discovery Rate (FDR) was set to 5%, and adjusted *q*-values < 0.05 were considered statistically significant.

### Transcriptional profiling

For microarray analysis ureters were manually dissected out of whole urogenital systems from differently staged embryos. Care was taken to clip off the proximal end at the kidney-ureter junction and the distal end just above the entry into the bladder. Visual inspection warranted the exclusion of contaminating tissues in these ureter preparations. We used fourteen or more ureters for each pool of male or female control or mutant E14.5 embryos (*n* = 2), 23 or more ureters from E11.5 (*n* = 2) or E12.5 (*n* = 4) control or IWP-2 (10 µM) treated explants, and 28 or more ureters from E12.5 (*n* = 2) control or BIO (10 µM) treated explants. Total RNA from each pool was extracted using either peqGOLD RNApure (#30-1010, VWR International GmbH, Darmstadt, Germany) or RNeasy micro kit (#74004, Qiagen, Hilden, Germany) and subsequently processed by the Research Core Unit Transcriptomics of Hannover Medical School. Agilent whole Mouse Genome Oligo v2 (4 × 44 K) microarrays (#G4846A; Agilent Technologies Inc., Santa Clara, CA, USA) were used for transcriptome analysis. Normalized expression data were filtered using Microsoft Excel.

For bulk RNA-Seq analysis, total RNA was extracted from E12.5 ureter explants treated for 18 h with 15 mM LiCl and water controls (*n* = 3) using the RNeasy Micro Kit (Qiagen). Library preparation (poly-A enrichment, stranded cDNA synthesis), quality control, and 150 bp paired-end sequencing on an Illumina NovaSeq platform were performed by Novogene (Munich, Germany). Raw reads were quality-filtered by Novogene to remove adapters and low-quality reads. Clean reads were aligned to the mouse reference genome (GRCm39) using HISAT2, and gene counts were generated with featureCounts. Differential gene expression analysis was performed using DESeq2 in R. Differentially expressed genes (DEGs) were defined by an adjusted *p*-value (padj) < 0.05 and an absolute log2 fold change (|log2FC|) > 1.

Functional enrichment analysis for up- and downregulated genes was performed with the subprogram “GO Biological Process 2025” within the web-based “Enrichr” gene list enrichment analysis tool (https://maayanlab.cloud/Enrichr/) [26–28] or with the DAVID 6.8 web software tool (david.ncifcrf.gov). Terms were selected based on *p* values. Microarray data were submitted to the Gene Expression Omnibus (GEO, http://www.ncbi.nlm.nih.gov/geo/) (GSE302034, GSE302036, GSE302032, GSE330379, GSE331377).

### In silico ChIP-seq analyses for CTNNB1

Publicly available TF ChIP-seq datasets for mouse (mm10) CTNNB1, TCF7, TCF7L1, TCF7L2 and LEF1 were downloaded from the database ChIP-Atlas (http://chip-atlas.org) using the “Peak browser” function with default settings (Table S31). CTNNB1 peaks that overlapped with at least one peak of either TCF7, TCF7L1, TCF7L2 or LEF1, respectively, were identified with the University of California Santa Cruz (UCSC) Genome Browser Data integrator tool (https://genome.ucsc.edu) [68] using default settings; non-overlapping CTNNB1 peaks were discarded. In the web-based analysis platform Galaxy Genome Annotation (https://annotation.usegalaxy.eu) [69] multiple overlapping CTNNB1 peaks from different experiments were merged into clusters using the bedtools MergeBED function [70] and annotated and assigned to nearest transcriptional start sites (TSS) with ChIPSeeker 1.28.3 [71] using default settings and the reference genome file mm10.ncbiRefSeq.gtf. Peak data and ENCODE candidate cis-regulatory elements (cCRE) (https://www.encodeproject.org) [72, 73] were visualized using the Integrative Genome Viewer (IGV) 2.19.4 [74]. Assignment of CTNNB1 peaks to differentially expressed genes from microarrays was performed in Microsoft Excel (Table S32).

### Reverse transcription-quantitative polymerase chain reaction (RT-qPCR)

RNA extraction and RT-qPCR analysis were performed on three pools each of 15 or more E12.5 ureters cultured for 18 h with 10 µM IWP-2 without FCS in the culture medium. Total RNA was isolated using the RNeasy micro kit (Qiagen), and cDNA was synthesized from a maximum of 2.5 µg of total RNA using RevertAid H Minus reverse transcriptase (EP0452, Thermo Fisher Scientific, Waltham, MA, USA) as described previously [75]. The NCBI tool Primer3 version 4.1 was used to design specific primers (Table S35) [76, 77]. RT-qPCR of mouse genes was performed in 10 µl of diluted qPCRBIO SyGreen Lo-ROX mix (PB20.11-01, PCR Biosystems Ltd., London, UK) with 400 nM primers and 1 ng/µl cDNA (final concentration) using a QuantStudio3 PCR system fluorometric thermal cycler (Thermo Fisher Scientific). Each of the three biological replicates represents the average of four technical replicates. The data were processed by the Relative Quantification Analysis Module (version 4.3, Applied Biosystems™ Analysis Software, Life Technologies Corp., Carlsbad, CA, USA) using relative quantification by the comparative threshold cycle (ΔΔCT) method with *Gapdh* and *Ppia* as reference genes [19]. Statistical analysis was performed using the unpaired, two-tailed Student’s *t* test (for *Axin2* with Welch’s correction) (GraphPad Prism version 7.03, GraphPad Software, San Diego, CA, USA; Microsoft Excel). The values are expressed as the means ± s.d. *P* < 0.05 was considered significant.

### Image documentation

Sections, cultures and stained whole mount specimen were photographed using a DM5000 microscope (Leica Microsystems, Wetzlar, Germany) with a Leica DFC300FX and a Leica K3C digital camera, a Leica DMI6000B microscope with a Leica DFC350FX and a Leica K3M digital camera, or a Leica Z6 APO microscope with a Leica DFC420C digital camera. All images were assembled in Adobe Photoshop CS4.

## Supplementary Information

Supplementary Material 1.

Supplementary Material 2.

## Data Availability

The datasets supporting the conclusions of this article are included within the article and its additional files. Microarray data have been deposited in the Gene Expression Omnibus under accession numbers GSE302034, GSE302036, GSE302032, GSE330379 and GSE331377.

## Abbreviations

avgFC: Average fold change
BIO: GSK-3 Inhibitor IX, 6-bromoindirubin-3-oxime
BMS493: Inverse pan-retinoic acid receptor (RAR) agonist
bp: Base pair(s)
CAKUT: Congenital anomalies of the kidney and urinary tract
DAPI: 4′,6-Diamidino-2-phenylindole
DAVID: Database for Annotation, Visualization, and Integrated Discovery
DFG: Deutsche Forschungsgemeinschaft
DMEM: Dulbecco's modified eagle's medium
DMSO: Dimethylsulfoxid
E: Embryonic day
ECM: Extracellular matrix
EGFP: Enhanced green fluorescent protein
ERT2: Modified ligand-binding domain of the human estrogen receptor
FC: Fold change
FCS: Fetal calf serum
FDR: False discovery rate
GEO: Gene expression omnibus
GFP: Green fluorescent protein
GO: Gene ontology
HE: Hematoxylin and eosin
IGV: Integrative genomics viewer
IRES: Internal ribosome entry site
IWP-2: Inhibitor of Wnt production-2
K: Kidney
KEGG: Kyoto Encyclopedia of Genes and Genomes
KO: Knockout
ND: Nephric duct
NMRI: Naval Medical Research Institute outbred mice
OE: Overexpression
P: Postnatal
PBS: Phosphate-buffered saline
PCR: Polymerase chain reaction
PFA: Paraformaldehyde
RA: Retinoic acid
RT-qPCR: Reverse transcription-quantitative PCR
SMC: Smooth muscle cell
TA: Tunica adventitia
TF: Transcription factor
TNB: TNB blocking buffer
TSA: Tyramide signal amplification
TSS: Transcriptional start sites
U: Ureter
UB: Ureteric bud
UCSC: University of California Santa Cruz
UE: Ureteric epithelium
UM: Ureteric mesenchyme
