# Supplementary material for: Mesenchymal WNT signaling coordinates epithelial and mesenchymal differentiation in the developing murine ureter
Source: Cell Commun Signal. 2026 Jul 21;24:413. doi: 10.1186/s12964-026-03094-6 (PMC13386783; doi:10.1186/s12964-026-03094-6)
Supplement: Supplementary file 1 — Supplementary Material 1. [file 12964_2026_3094_MOESM1_ESM.pdf]

Supplementary Figures

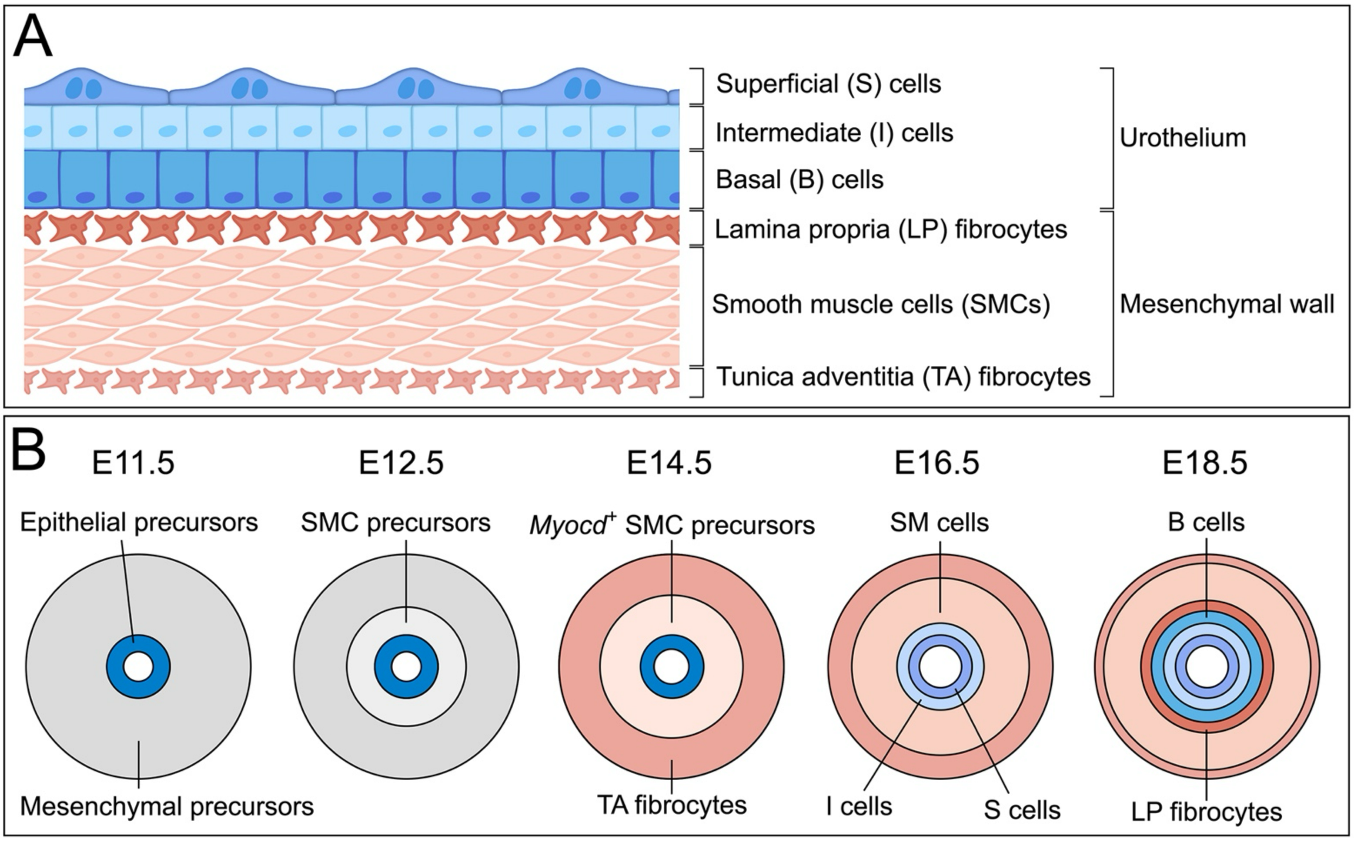

Figure S1. The organization of the cell layers in the epithelial and mesenchymal tissue compartments of the mouse ureter and its development. (**A**) Scheme of the cell layers in the epithelial tissue compartment (the urothelium) and the surrounding mesenchymal wall of the mature ureter in the mouse. (**B**) Scheme of the staggered development of the cell layers within the epithelial and mesenchymal tissue compartment of the ureter from E11.5 to E18.5. Abbreviations used in (**B**) are introduced in (**A**).

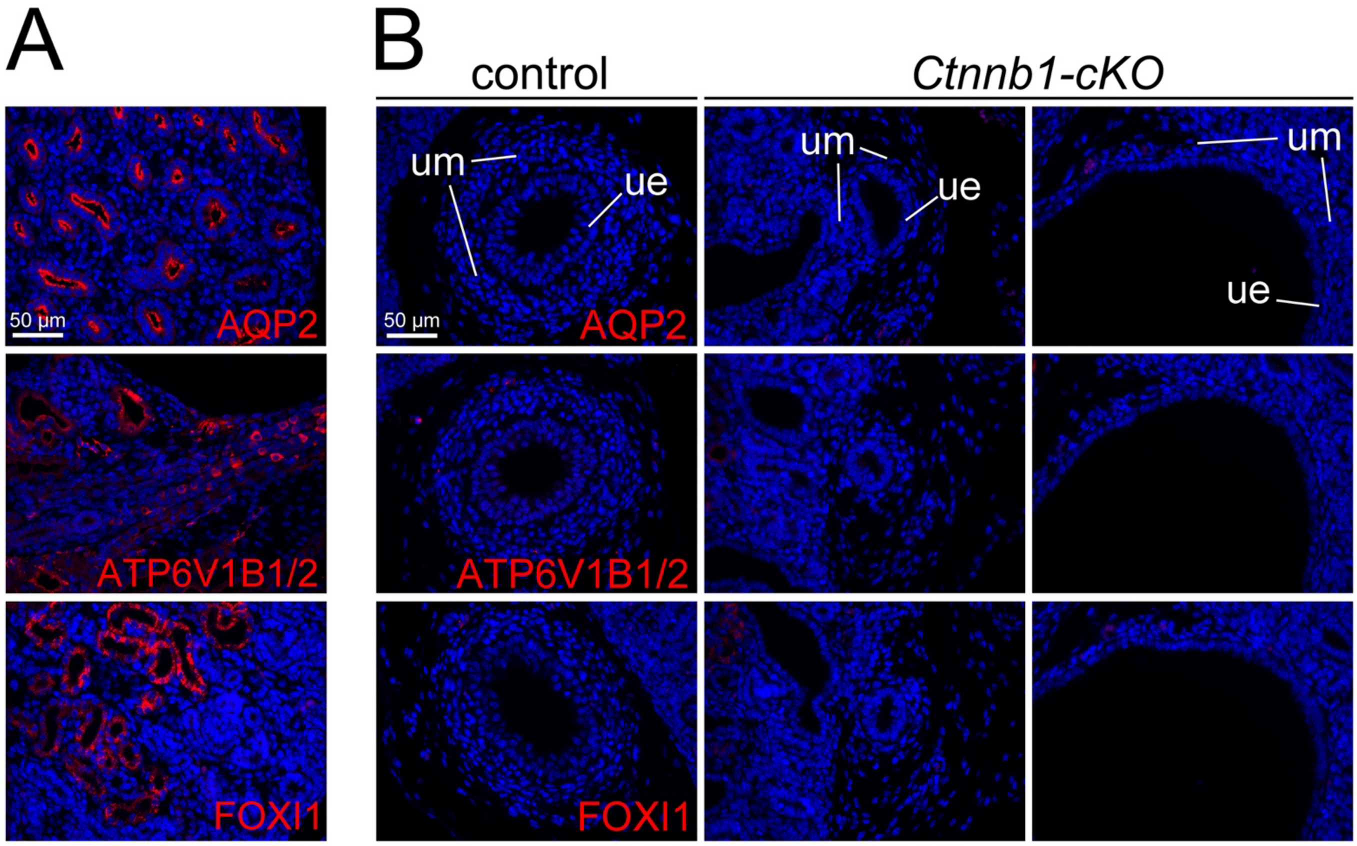

Figure S2. Markers of collecting duct principal and intercalated cells are not expressed in E18.5 *Ctnnb1-cKO* ureters. (**A, B**) Immunofluorescence analysis of sagittal sections of normal E18.5 kidneys (**A**) and transverse sections of E18.5 control and *Ctnnb1-cKO* ureters (**B**) for expression of markers of principal cells (AQP2) and intercalated cells (ATP6V1B1/2, FOXI1) in the collecting duct system. Note that a rare undilated specimen was used in addition to the frequent dilated specimens for the E18.5 *Ctnnb1-cKO* ureters. *n*=4 per probe. ue, ureteric epithelium; um, ureteric mesenchyme.

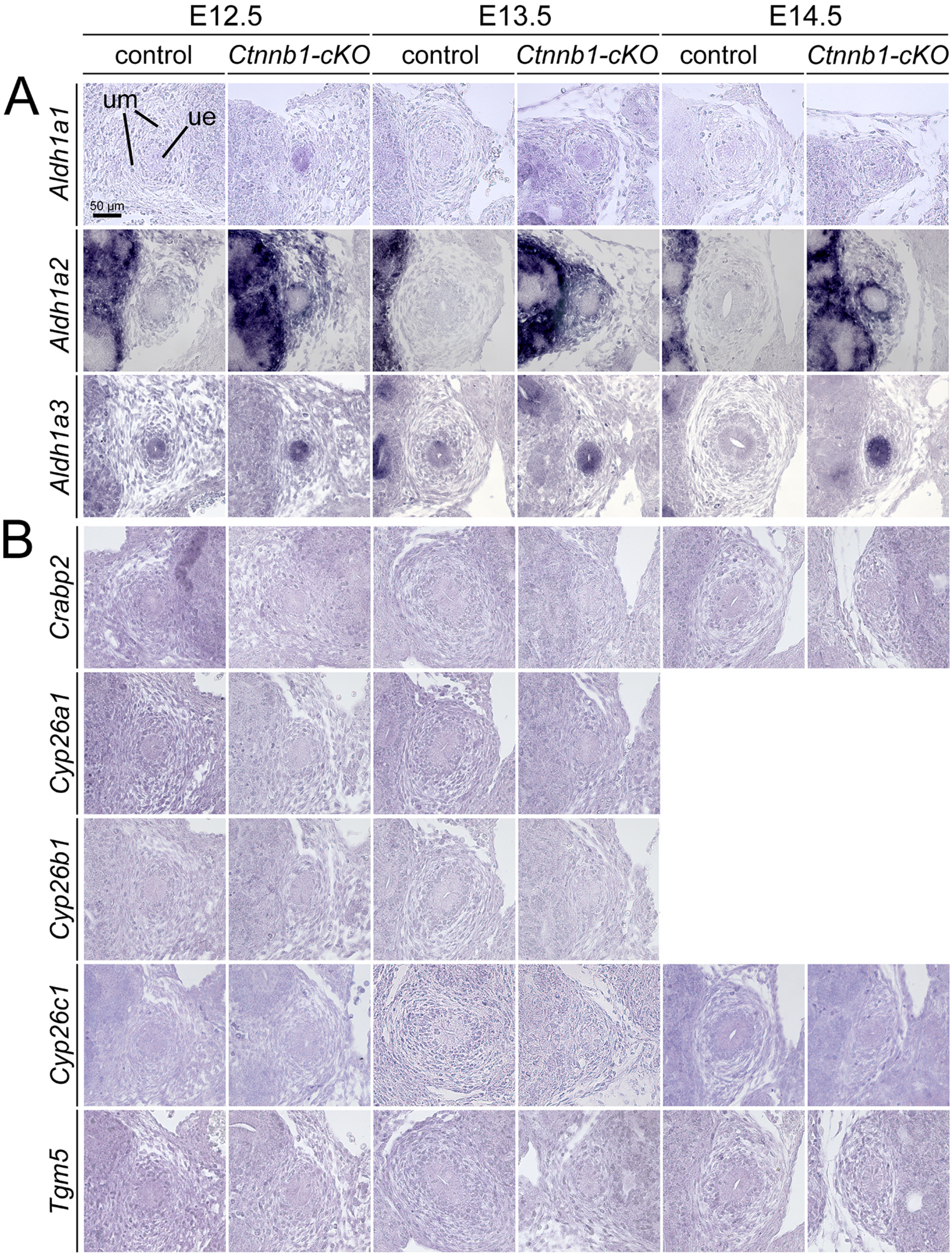

Figure S3. Expression of some retinoic acid (RA) signaling components is affected in *Ctnnb1-cKO* ureters. (**A, B**) RNA *in situ* hybridization analysis of proximal ureter sections from control and *Ctnnb1-cKO* embryos at E12.5, E13.5 and E14.5 to detect the expression of genes that encode RA biosynthetic enzymes (**A**), and RA-responsive genes (**B**). *n*=3 per probe, stage and genotype. ue, ureteric epithelium; um, ureteric mesenchyme.

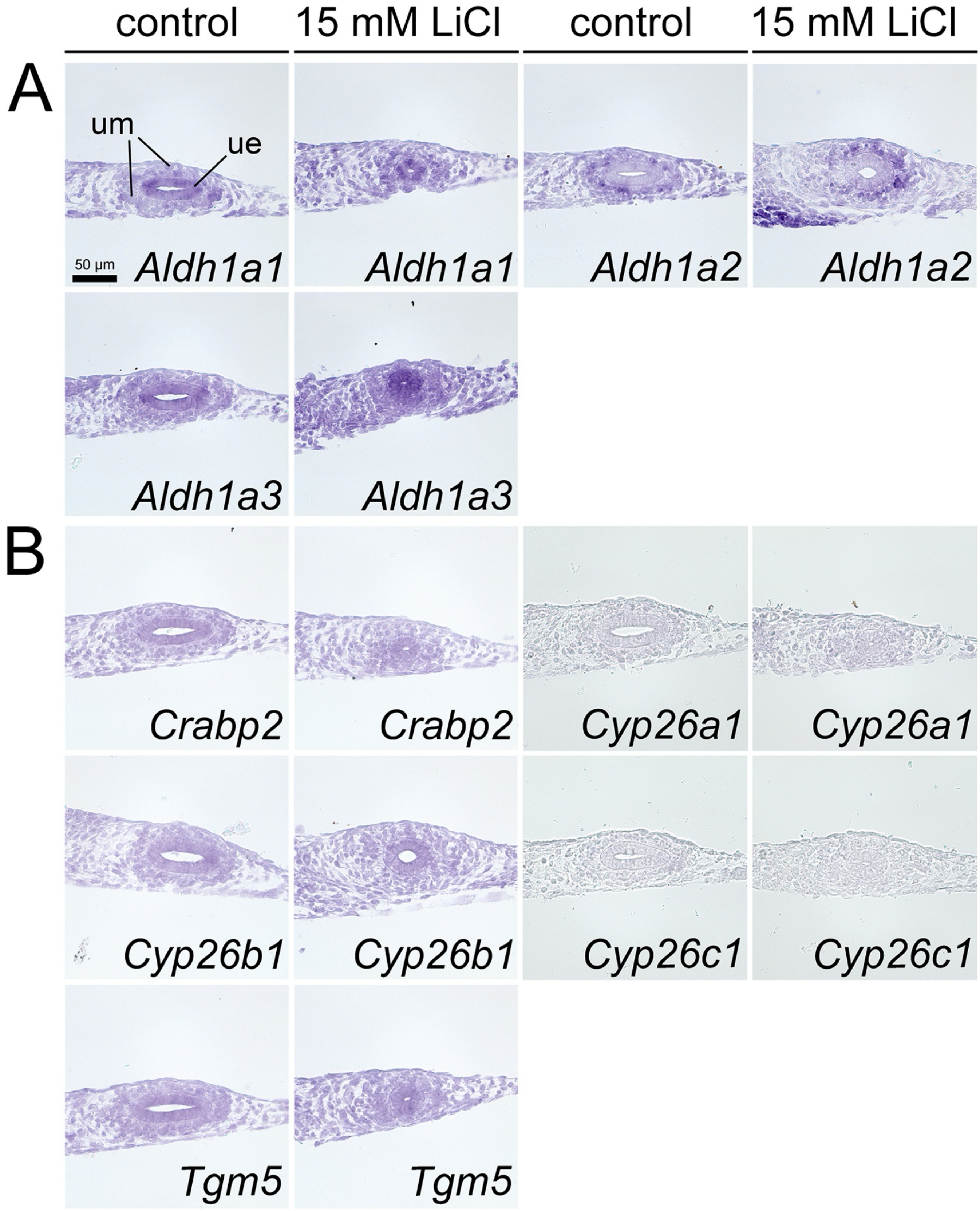

Figure S4. LiCl treatment of early ureter explants increases retinoic acid (RA) production and RA signaling. (**A, B**) RNA *in situ* hybridization analysis of the expression of genes encoding RA biosynthetic enzymes (**A**) and RA-responsive genes (**B**) in proximal sections of wild-type E12.5 ureter explants cultured for two days with 15 mM LiCl. ue, ureteric epithelium; um, ureteric mesenchyme.

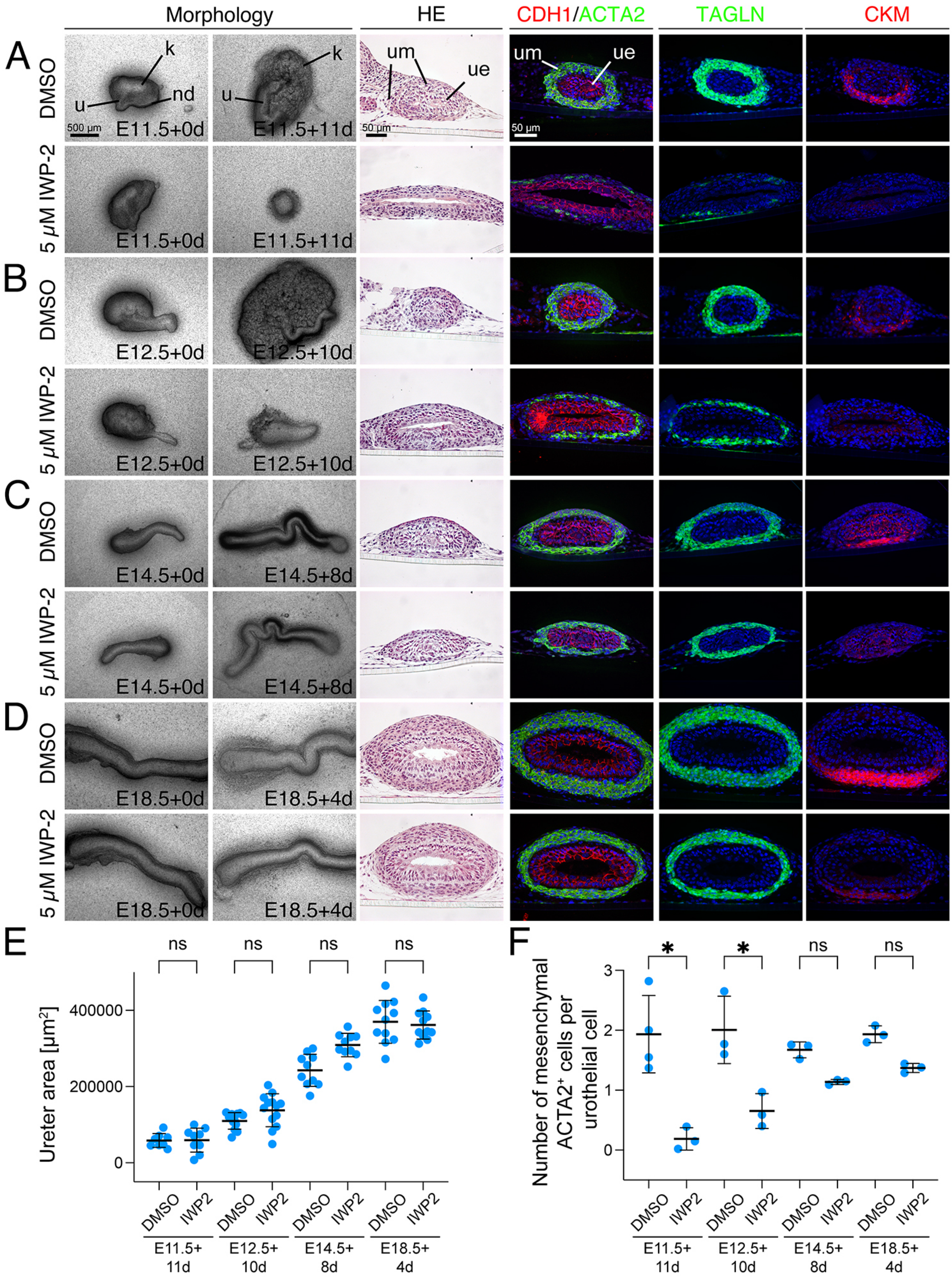
Morphology
HE
CDH1/ACTA2
TAGLN
CKM
A
B
C
D
DMSO
5 μM IWP-2
k
u
nd
um
ue
500 μm
50 μm
E11.5+0d
E11.5+11d
E12.5+0d
E12.5+10d
E14.5+0d
E14.5+8d
E18.5+0d
E18.5+4d
E
ns
Ureter area [μm²]
400000
200000
0
DMSO
IWP2
E11.5+ 11d
E12.5+ 10d
E14.5+ 8d
E18.5+ 4d
F
*
Number of mesenchymal ACTA2+ cells per urothelial cell
3
2
1
0

Figure S5.

Figure S5. Mesenchymal WNT signaling is required in the early undifferentiated ureter for smooth muscle cell (SMC) development. Wildtype ureters were isolated at E11.5 (**A**), E12.5 (**B**), E14.5 (**C**) and E18.5 (**D**), and cultured with either solvent (DMSO) or with 5 µM IWP-2 for a variable number of days until reaching a common end-point. The ureters were examined for morphology at the beginning and the end of the culture period (Morphology, column 1 and 2), for histological appearance using hematoxylin and eosin staining (HE, column 3), and for expression of the epithelial marker CDH1 and the smooth muscle cell markers ACTA2, TAGLN and CKM at the end of the culture period (column 4 to 6). *n*=3 for each assay. k, kidney; nd, nephric duct; u, ureter; ue, ureteric epithelium; um, ureteric mesenchyme. (**E, F)** Quantification of the ureter area (**E**) and of the $ACTA2^{+}$ cells (**F**) in the ureter cultures shown in (**A-D**), $n \geq 4$. Statistical data are presented as mean±standard deviation. Statistical significance was determined by a Kruskal-Wallis test followed by the Benjamini-Krieger-Yekutieli two-stage linear step-up procedure for multiple comparisons (FDR = 5%). Asterisks (*) indicate a discovery ($q < 0.05$); ns, not significant. See Table S19 and S20 for source data and statistics.

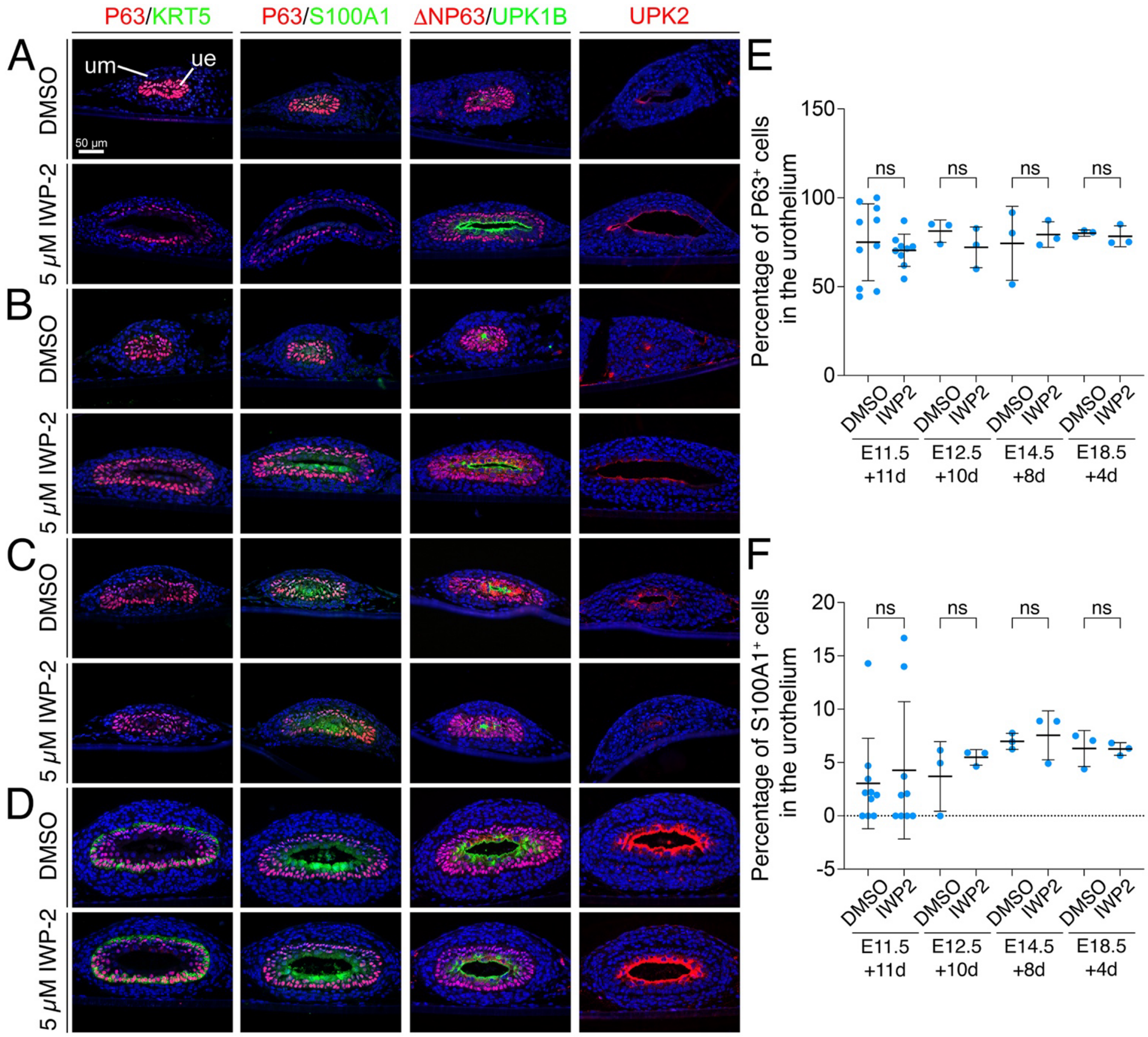

Figure S6. Pharmacological inhibition of WNT signaling in ureter explant cultures does not affect epithelial stratification and differentiation. (A-D) Wildtype ureters were isolated at E11.5, E12.5, E14.5 and E18.5, and cultured with either solvent (DMSO) or with 5 µM IWP-2 for 11 days (E11.5 explants, **A**), 10 days (E12.5 explants, **B**), 8 days (14.5 explants, **C**) and 4 days (18.5 explants, **D**), respectively, to reach a common endpoint. Immunofluorescence analysis was performed at the culture endpoint for markers of epithelial stratification and I cell differentiation (P63, ΔNP63) and S cell differentiation (S100A1, UPK1B, UPK2). (**E, F)** Quantification of the percentage of P63^{+} cells (**E**) and of S100A1^{+} cells, respectively (**F**). $n \geq 3$. Statistical data are presented as mean±standard deviation. Statistical significance was determined by a Kruskal-Wallis test followed by the Benjamini-Krieger-Yekutieli two-stage linear step-up procedure for multiple comparisons (FDR = 5%). Asterisks (*) indicate a discovery ($q < 0.05$); ns, not significant. See Table S21 for source data and statistics. ue, ureteric epithelium; um, ureteric mesenchyme

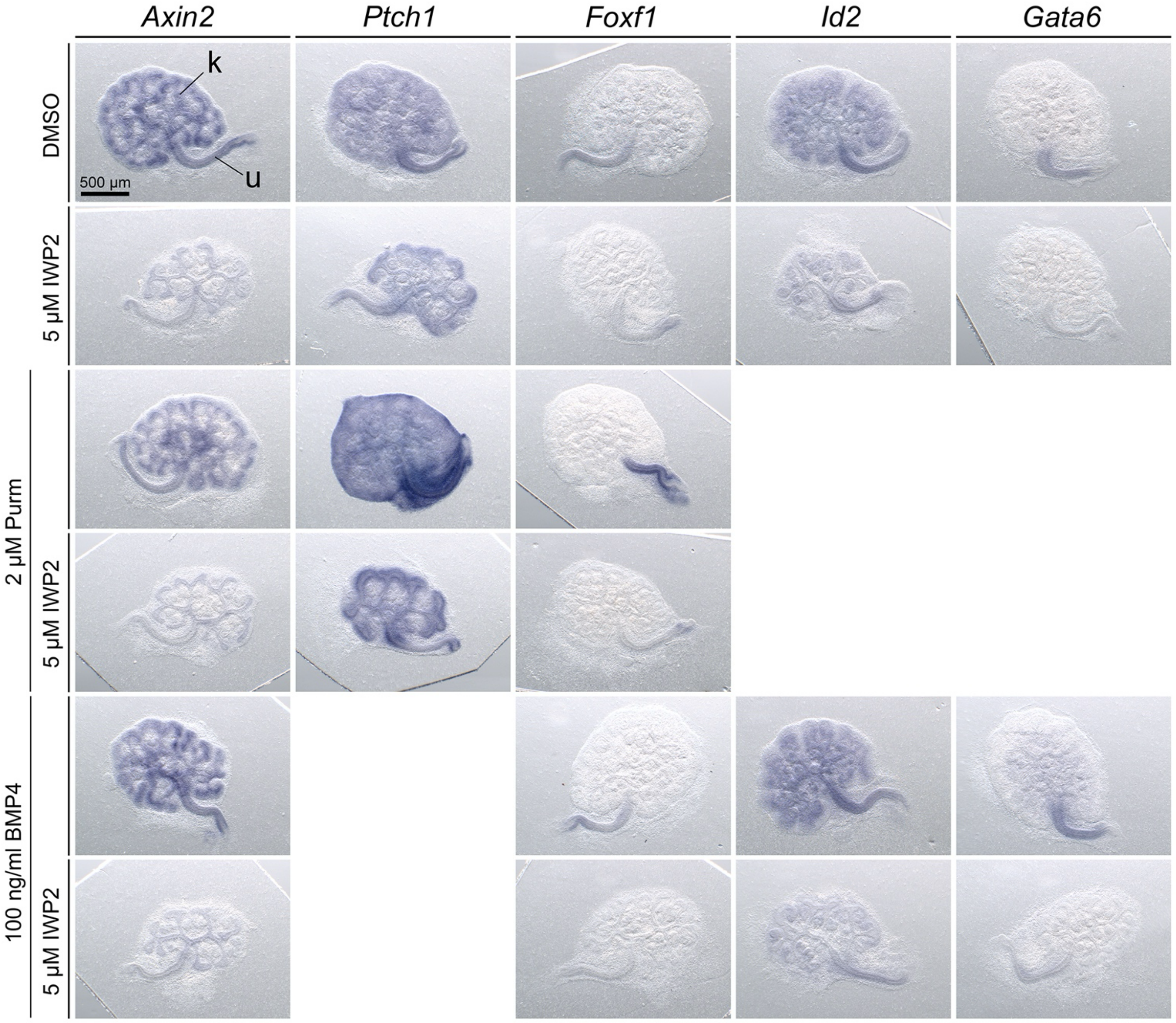

Figure S7. WNT signaling affects the expression of components of the SHH-FOXF1-BMP4 signaling axis in the UM differently. E12.5 ureters were cultured for 2 days in the presence of DMSO or 5 µM IWP-2, either with or without the HH signaling activator purmorphamine (2 µM), and either with or without 100 ng/ml BMP4. The specimens were then subjected to whole-mount RNA *in situ* hybridization to analyze the expression of target and effector genes of WNT signaling (*Axin2*), SHH signaling (*Ptch1, Foxf1*) and BMP signaling (*Id2, Gata6*). *n*=6. k, kidney; u, ureter.
